# Joint Income-Wealth Inequality: Evidence from Lucerne Tax Data

**DOI:** 10.1007/s11205-022-02887-9

**Published:** 2022-02-24

**Authors:** David Gallusser, Matthias Krapf

**Affiliations:** 1 University of Basel, Basel, Switzerland; 2 University of Lausanne, University of Basel and CESifo, Basel, Switzerland

**Keywords:** Inequality, Income, Annuitized wealth, Tax data

## Abstract

Using tax data from the Swiss canton of Lucerne, we study how measures of economic inequality change if they account for income and wealth rather than income alone. Joint income-wealth, the sum of labor income and annuitized wealth, serves as a measure of combined inequality of income and wealth. Inequality measured using joint income-wealth is higher than measured using income alone. We refine existing annuitization techniques by introducing heterogeneous returns. The joint distribution of labor income and annuitized wealth displays strong tail dependence at the top and a negative association for negative annuitized wealth. A decomposition shows that the underlying marginal distributions of labor income and annuitized wealth account for most of joint income-wealth inequality, whereas their association matters only in the tails.

## Introduction

Income is not the only financial resource available to households. People’s material well-being also depends on their stock of wealth. They can use their financial assets to finance their consumption and they can borrow against real assets such as housing wealth. A combined measure of income and wealth, thus, allows to assess inequality of consumption possibilities more comprehensively than one based only on income. Although there is an emerging literature on wealth inequality, we still know far less about the wealth distribution than about the income distribution. Our knowledge of the joint distribution of income and wealth is even more limited, as most existing studies only look at either income or wealth inequality. How does our assessment of inequality change if we account for income and wealth at the same time?

In this study, we exploit a data set that covers the universe of taxpaying households in this Swiss canton of Lucerne over the years 2005–15. Given that Switzerland is one of few countries besides Norway (Halvorsen and Thoresen 2021; Fagereng et al. 2020) and Spain (Durán-Cabré et al. 2019) that still have a wealth tax, we observe administrative information on both income and wealth.

We use the concept of annuitized wealth to convert wealth stocks into an item that is comparable to income flows. The concept was originally developed by Weisbrod and Hansen (1968) and has been mainly applied in poverty research (e.g. van den Bosch 1998; Short and Ruggles 2005; Zagorsky 2005; Brandolini et al. 2010; Azpitarte 2012). However, Rendall and Speare (1993) or Wolff and Zacharias (2009) also use annuitized wealth to measure inequality of “sustainable consumption” among a broader population.

Annuitized wealth measures how much somebody could sustainably consume if they were to reduce their wealth stock to zero by the end of their expected remaining lifetime. The sum of labor income and annuitized wealth, which we call *joint income-wealth* following Kuypers and Marx (2018), provides a more complete picture of consumption possibilities than regular income consisting of labor income and disbursed capital income (i.e. the sum of interest, dividends, and rent).

We further expand the concept of annuitized wealth to allow for variation in returns across different asset classes and along the distribution of financial assets. By introducing heterogeneous returns, we address a shortcoming of the previous literature, notably the so-called “capitalization method” (Saez and Zucman 2016), which largely ignored that not all wealth is productive capital (McGrattan 2015). Our procedure accounts for recent evidence that wealthier people tend to be able to generate higher returns to their assets (Bach et al. 2020; Fagereng et al. 2020).

We do not only study the distribution of aggregate joint income-wealth but also investigate the dependence of labor income and annuitized wealth. In particular, we analyze the association between the two financial resources throughout the entire distribution and measure its contribution to inequality. Our analysis contributes to the nascent literature on the joint distribution of income and wealth that focuses mainly on the incidence of the jointly income rich and asset rich such as Roine and Waldenström (2008), Aaberge et al. (2018), Fisher et al. (2017), or Berman and Milanovic (2020). One notable exception is Jäntti et al. (2015) who find a positive association between income and wealth throughout the distribution and across countries using survey data.

In contrast to Jäntti et al., we are able to draw on an administrative data set with high-quality information on wealth. This feature of the Swiss setting has so far been touched upon by few studies in different contexts (Foellmi and Martínez 2017; Krapf 2018). We are, however, the first to use Swiss data to estimate annuitized wealth and its dependence on labor income.

While the Swiss constitution obliges the 26 cantons to apply a tax on net wealth, the cantons are largely autonomous in how they implement this tax and in how they assess the value of many assets, in particular real estate and non-listed firms. In a related exercise, Martínez (2020) collects tax data for several Swiss cantons and presents a detailed account of the composition of wealth across the distribution and the joint distribution of income and wealth. While Martínez’s study gives an assessment of the dependencies between income flows and wealth stocks, we examine to what extent these dependencies translate into unequal consumption possibilities. Moreover, using data for only one canton allows us to examine the link between labor income and annuitized wealth at a more micro level. Practices regarding, for example, the valuation of housing wealth still vary across cantons.

We chose the canton of Lucerne because it is highly representative of Switzerland as a whole. Brülhart et al. (2021) show that Lucerne is the canton whose top 1% wealth share is closest to that of the entire country. Statistics available from the Swiss Federal Tax Administration show that in 2015, mean net wealth per household in Lucerne at 341,000 CHF almost exactly equalled mean wealth per household in Switzerland at 342,000 CHF and mean taxable income per household in Lucerne at 56,000 CHF was relatively close to mean taxable income per household in the entire country at 59,000 CHF.^1^

Most of the existing literature on wealth inequality relies either on survey data (Kennickell 2009) or estimates wealth from estate tax data (Kopczuk and Saez 2004), from capital income data (Saez and Zucman 2016), or from rich lists (Vermeulen 2018). These previous studies, thus, could observe wealth only at a more aggregate level and, often, focused on the top of the distribution. Most importantly, our data allow us to document how (annuitized) wealth covaries with labor income, which is largely still a puzzle.

Our documentation of combined inequality of income and wealth proceeds in two steps. Following, for example, Brandolini et al. (2010) and Kuypers and Marx (2018), we first investigate the joint distribution of labor income and annuitized wealth. In the second step, we examine distributional properties of joint income-wealth, the sum of labor income and annuitized wealth.

In the first step, we estimate non-parametric copulas by quantifying and visualizing $100 \times 100$ association matrices between percentile rank pairs. This approach differs from Brandolini et al. and from Kuypers and Marx, who measure the share of jointly income-poor and asset-poor households.^2^ Our methodology is more closely related to Aaberge et al. (2018) and to Chetty et al. (2017). An important previous study in this literature is Jäntti et al. (2015), who parametrically model the joint distribution of income and wealth using a mixture model, in which they distinguish between negative wealth, zero wealth, and positive wealth. While the data that are available to us allow us to relax their parametric assumptions, distinguishing between negative, zero, and positive wealth remains important.

Like previous literature on the relationship of income and wealth, we find strong tail dependence between labor income and annuitized wealth. The top 1% of the annuitized wealth distribution are very likely to be among the top 1% of the labor income distribution. The positive dependence between labor income and annuitized wealth is less pronounced in parts of the distribution further away from the tails. Among younger individuals, who have low levels of wealth, and among older individuals, who receive little labor income, we observe only a weak association.

More strikingly, we show that the correlation between labor income and annuitized wealth switches signs and becomes negative for negative annuitized wealth. This relationship is driven by a positive association between debt and high labor income. We thus provide a more detailed documentation of a pattern that has been found in other settings. Rios-Rull and Kuhn (2016, Table 8), for example, document that households with negative net wealth tend to earn relatively high incomes in the Survey of Consumer Finances. Krapf (2018) finds the same phenomenon in tax data from the Swiss canton of Bern. Krapf shows not only that the correlation between wealth and income turns negative for negative net wealth, but also that taxpayers with negative net wealth experience large gains on average in both income and wealth in subsequent years.

In the second step, we examine joint income-wealth, i.e. the sum of labor income and annuitized wealth, and compare it to current income, i.e. the sum of labor income and disbursed taxable capital income. We find that joint income-wealth is distributed more unequally than taxable income. The difference between joint income-wealth inequality and current income inequality is most pronounced for individuals aged 65 and older, who have the highest levels of annuitized wealth, but it is not limited to this age group.

We then apply a decomposition method by Rothe (2015) to distinguish between the contributions of the underlying marginal distributions of labor income and annuitized wealth and their dependence to joint income-wealth inequality. The greater inequality of joint income-wealth is mostly due to annuitized wealth being higher, on average, than taxable disbursed capital income, but similarly concentrated in the upper tail. The positive dependence between the two financial resources plays only a minor role, even for individuals shortly before retirement where the dependence is strongest.

Our results add to the literature on the evolution of inequality and its drivers. Reviewing the literature on wealth inequality, Benhabib and Bisin (2018) argue that the tail of the wealth distribution is determined by either the tail of the income distribution or by return heterogeneity, but not by both. Similarly, we find that joint income-wealth inequality is determined by labor earnings among younger generations and by annuitized wealth among older generations, but not by both because the association of the two factors only plays a minor role.

Our findings further support the view that measures of economic inequality need to account for wealth in addition to traditional measures of income, particularly because wealth is distributed more unequally than income and wealth stocks have become more important (Piketty and Zucman 2014). Related research by Brülhart et al. (forthcoming) shows that Swiss wealth taxes induce substantial behavioral responses and are thus an important determinant of the wealth distribution.

The remainder of this paper is organized as follows. Section 2 explains the concept of annuitized wealth and outlines the methodology we use to assess joint distributions. Section 3 provides an overview of the institutional setting and describes the data used in this study. In Sect. 4, we visualize the joint distributions between labor income and annuitized wealth, and describe the patterns we find. In Sect. 5, we examine the distribution of joint income-wealth before decomposing it into the contribution of its marginals distribution and their dependence in Sect. 6. Section 7 concludes.

## Analytical Framework

Most of the existing literature on inequality relies on a concept of current income (Brandolini et al. 2010), which comprises the sum of all income flows in a given period, usually a year. Current income includes capital income flows and is, hence, not independent of wealth. Thus, we can express *current income* $Y_c$ as the sum of two factor incomes

1 $$\begin{aligned} Y_c = Y_l + rW_N, \end{aligned}$$

where the $Y_l$ captures all labor income, including earnings from employment, pensions, and transfers, $W_N$ is net wealth, the difference between the sum of all assets and total debt, and *r* is the return on net wealth. Current income as a measure of economic well-being ignores that wealth allows people to also consume their stock of wealth on top of just their returns. In this paper, we define income as income after contributions to social insurances and taxable transfers but before income and wealth taxes (see discussion in Sect. 3).

Against this background, Weisbrod and Hansen (1968) developed their concept of annuitized wealth. They suggest to annuitize wealth into a hypothetical income stream assuming that households receive not only returns on their assets but consume their wealth stock over their expected remaining life time

2 $$\begin{aligned} Y_a^{N} = \left[ \frac{r}{1-(1+r)^{-n}}\right] \cdot W_{N}, \end{aligned}$$

where *n* is remaining life expectancy and, thus, the expected duration of the annuity. A shorter remaining life expectancy or a higher interest rate would increase the value of the annuity.

In this paper, we apply Weisbrod and Hansen’s annuitization formula not to the overall stock of net wealth, but distinguish between different asset classes, which may generate different rates of return. Following Fagereng et al. (2020) and Bach et al. (2020), we allow for variation in return rates not only across asset classes but also within the class of financial assets. The resulting measure of annuitized wealth $Y_a$ takes the form

3 $$\begin{aligned} Y_a = \left[ \frac{r_f^p}{1-(1+r_f^p)^{-n}}\right] \cdot W_f + \left[ \frac{r_h}{1-(1+r_h)^{-n}}\right] \cdot W_h - \left[ \frac{r_d}{1-(1+r_d)^{-n}}\right] \cdot D, \end{aligned}$$

where $r_f^p$ is a percentile-specific return on financial assets $W_f$, $r_h$ is the return on housing wealth and real estate $W_h$, and $r_d$ is the interest rate on debt *D*.

Finally, we take the sum of annuitized wealth and labor income to get our aggregate measure of economic well-being

4 $$\begin{aligned} Y_j = Y_l + Y_a, \end{aligned}$$

that we call *joint income-wealth* following Kuypers and Marx (2018). Joint income-wealth as a measure of consumption possibilities accounts for accrued capital gains, which are not part of current income.^3^

The advantages of annuitized wealth compared to other measures of capital income and wealth, however, come at a cost (see Brandolini et al. 2010; Kuypers and Marx 2018). First, annuitization imposes structure on the measurement of inequality. The required assumptions about the annuities’ length and interest rates particularly affect the relative position of the elderly. Second, we use annuities in the framework of this paper as a hypothetical measure of yearly consumption possibilities rather than actual income streams over the expected remaining years of life.

While annuitization of wealth stocks is possible, we do not argue that households generally purchase annuities. Swiss households, in fact, rarely make use of annuitization. A recent literature argues that households should in fact purchase annuities to smooth consumption and to insure against longevity risk, though. Brown et al. (2021), for example, examine behavioral biases that may reduce people’s ability to correctly value annuities. While reverse mortgages are, in principle, available, relative illiquidity of housing wealth is another impediment to annuitization (Pashchenko 2013). On the other hand, while insurance companies generally tend to be better at managing longevity risk than their customers, an increasing number of pension plans are underfunded (Tang et al. 2010).

The aggregation of joint income-wealth could blur complex dependencies between labor income and annuitized wealth. This is why we will additionally assess the joint distribution of the latter two variables. Following Jäntti et al. (2015), we analyze the joint distributions by describing the respective marginal distributions and their dependence structure separately. This approach is motivated by Sklar’s theorem according to which we can express the joint distribution of two continuous variables with a unique copula function that connects the variable’s marginal distributions. The copula function is itself a joint distribution function of the two uniformly distributed rank variables, i.e.,

$$\begin{aligned} F_{Y_l, Y_a}(y_l, y_a)=C_{Y_l, Y_a}(F_{ Y_a}( y_a),F_{ Y_l}(y_l)), \end{aligned}$$

in the case of labor income and annuitized wealth. Like Aaberge et al. (2018) and Chetty et al. (2017), we discretely approximate the copula density by empirical association matrices that reflect the sample probabilities of every percentile rank combination. We will focus on the association matrices between labor income and annuitized wealth. We additionally provide the corresponding matrix between current income and net wealth in Appendix E.

Note that our variables of interest are not strictly continuous. They exhibit important mass points at zero as non-negligible parts of the population do either not receive any income or have zero net wealth. As a consequence, there is a range of quantile ranks linked to zero that renders it impossible to attribute these ranks uniquely to another variable’s rank. In other words, we cannot infer a uniquely defined copula across mass points. We circumvent the problem of indeterminacy by assuming the ranks at mass points to be uniformly distributed.

## Data

Our data cover the universe of tax returns in the Swiss canton of Lucerne between 2005 and 2015. Every resident aged 18 and older must submit a tax declaration independently of whether their income or wealth are below any minimum thresholds. In total, we have access to 2.36 million entries. A key feature of our data is that they contain not only income but also wealth. We also observe most items that wealth is composed of, such as debt, real estate and financial assets, and that income is composed of, such as income from self-employment or from dependent employment, interest, dividends, pensions and social insurance benefits. We also observe a number of items that are relevant for taxation, such as age and marital status, but not education and other socio-economic background variables. Brülhart et al. (forthcoming) use the same data set to assess behavioral responses to wealth taxation. Following Fagereng et al. (2020), we pool our data over the sample period 2005 to 2015 to improve statistical power. We will, however, also show results for a single sample year in the appendix.

Swiss tax authorities treat married as well as registered same-sex couples as one entity.^4^ On the other hand, unmarried and cohabiting couples are treated as two separate tax units. While we have information about dependent children, we do not know if they share the same household.

As we lack reliable information on the household structure, we compare income and wealth across individuals independently of the household composition. We divide all variables for married couples by two and assign one half to each spouse. Thereby, we imply that economic resources within couples are shared equally. This is a strong but nonetheless conservative assumption as it tends to underestimate inequality (see e.g. Piketty et al. 2018, pp. 590–94). After splitting couples we end up with 3.22 million observations.

For couples, our approach corresponds to a “per-adult" equivalence scale, implying that, independently of the presence of children, couples require as much income as the two partners would if they were singles to attain the same consumption level. This is different from the equivalence scales that the literature on income inequality employs to assess individual consumption possibilities. Our approach, however, is in line with the literature on wealth inequality. Cowell and van Kerm (2015) argue that in the context of wealth inequality, equivalence scales would have to account for future household structure on top of current household structure. Jäntti et al. (2015) conclude that it is largely an unsettled question whether it is appropriate whether to use equivalence scales when analyzing wealth inequality. In addition, our data only provide limited information on household size, for example we can only identify couples if they are married.

Note that to assure anonymity, all variables in the raw data are truncated from above. Stock variables are truncated at 40 million Swiss francs and flow variables are truncated at 2 million Swiss francs. We have information on the means of the truncated variables over all individuals to whom truncation applies in a given year and we use these yearly means instead of the unobserved true values. Since we only analyze distributions across percentiles and the truncation is always far above twice the lower bound of the top percentile, the truncation from above is not problematic for our analysis even though we divide wealth and income by two for married couples.

Our data have the advantage of providing information on wealth across the entire distribution.^5^ Throughout this paper, wealth will be defined as household net wealth, which we measure using the data item *Reinvermögen*, rather than gross wealth or assets. The tax administration computes *Reinvermögen* by subtracting household debt from total household wealth if the difference between the two is positive and sets it to zero otherwise. We also observe gross wealth and debt separately and replace raw net wealth with gross wealth minus debt if raw net wealth equals zero and debt is greater than gross wealth.^6^ Gross wealth includes everything a taxpayer owns, such as cash, financial assets, real estate located in Lucerne, shares of non-incorporated firms or cars.^7^ The wealth components, real estate, financial assets and business wealth, that we use to impute returns on average add up to 96% of gross wealth. We assume the remainder is wealth that does not yield returns such as cars.

Given the nature of our data, we observe earnings only *after* contributions to social insurances. As noted above, we also take all taxable transfers into account. In Switzerland, most government transfers, including pensions, unemployment benefits and maternity benefits, are part of taxable income, while contributions on earnings to these social insurances are tax exempt. We do not observe social assistance and assistance for the elderly and disabled (*Ergänzungsleistungen*) since they are not taxable.

Yet, we analyze *pre-tax* information on both income and wealth. We would not expect to find very different results if we looked at post-tax outcomes, in particular because the tax schedules in Lucerne are not very progressive. Note, however, that, in Switzerland, tax burdens vary across municipalities, which can set different multipliers that will be applied to the cantonal schedules. Roller and Schmidheiny (2016) show that this has implications for sorting: high-income households tend to settle in low-tax municipalities, which, in turn, tends to drive up living costs in these low-tax municipalities.

The only items that households do not have to report to the tax administration are household inventory and pension savings in employer covered or private pension plans. After retirement, taxpayers have the choice to either cash-out their pension savings, in which case they become taxable wealth, or to have them paid out in the form of monthly installments over the rest of their life, in which case they become taxable income (Bütler and Ramsden 2017). Wealth would be distributed less unequally if we could include pension savings. Simulations by Foellmi and Martínez (2017), however, show that including them would not fundamentally change the distribution.^8^

Households must collect and provide information on income from self-employment themselves, and submit statements on income from dependent employment or from pensions with their tax declaration. This, in combination with the banking secret, may induce underreporting (Alstadsæter et al. 2019).^9^ Households also report their financial assets themselves by submitting their own bank statements when filing their tax returns. There is, however, a 35% withholding tax that is applied to all income from interest and dividends. These tax payments are returned upon declaration of financial assets, which provides an incentive to report correctly. The only third-party reported component of wealth is real estate, which is assessed by the cantonal administration.

Note that cantons do not generally assess housing wealth at market values but apply a discount and rarely update real estate valuations.^10^ In the following, we will correct for the undervaluation of real estate by inflating real estate assets in the tax data by 150%. This inflation factor follows Brülhart et al. (2018), according to whom tax values of real estate in Switzerland correspond, on average, to two thirds of their market value.

There is also a discussion whether to account for illiquid assets like real estate in estimating annuitized wealth. In line with our discussion of the availability of reverse mortgages in Sect. 2, recent studies, however, find large consumption responses to house price changes (Berger et al. 2018). Unfortunately, our data do not allow us to distinguish between owner-occupied housing, which is likely most illiquid, and other real estate. To examine the importance of real estate, we will provide a robustness check, in which we do not inflate real estate values but use them as we observe them in our data. Research by Baselgia and Martínez (2020) suggests that real estate plays a substantial role as a driver of increasing wealth stocks in Switzerland.

We measure labor income and disbursed taxable capital income following Atkinson and Lakner (2017). They assign two thirds of income from self-employment to labor income and one third to capital income. Labor income, hence, is the sum of income from dependent employment, pension income, social insurance benefits and two thirds of income from self-employment. Capital income is the sum of income from interest, from dividends, from real estate and housing property as well as the remaining third of income from self-employment. All components of capital income can be negative, the item most likely to be negative is interest. We define current income as the sum of labor income and capital income (see Eq. 1).

Annuitized wealth $Y_a$ is stock-based as described in Eq. (3). It assumes different returns across asset classes and across percentiles of the distribution of financial assets that remain constant over time. Our computation of annuitized wealth exploits that we directly observe stocks of different assets. We construct $W_f$ from the sum of financial assets (“Wertschriftenvermögen”) and shares of non-incorporated firms (“Betriebsvermögen”) that we directly observe in our data for each taxpayer. To measure $r_f^p$, we rely on Fagereng et al. (2020)’s findings, which are for Norway but over the same period as in our data, 2005-15.

We use publicly available information by consultancy *Wüest Partner* for the area “Central Switzerland,” which Lucerne is part of, to assess real estate income and publicly available information from the Swiss National Bank (SNB) to assess interest rates on debt, resulting in $r_h = 3.90\%$ and $r_d = 2.78\%$. See Appendix A.B for further details on the sources and construction of these interest rates and returns. Finally, we also discuss the distribution of annuitized wealth using constant returns as originally proposed by Weisbrod and Hansen and described in Eq. 2. Following Kuypers and Marx (2018), we use interest rate $r=2\%$ for this exercise.

The Swiss Federal Statistical Office provides official figures on gender- and age-specific life expectancy for years 2008-13. To get a measure of *n*, we assign these official numbers to the individuals in our data based on their age and marital status. We use life expectancy of the female spouse for both partners of married couples. Following Brandolini et al. (2010) and Kuypers and Marx (2018), we can express $n=T_1+(T_2-T_1)/b$ for couples, where $T_1,T_2$ are the life expectancies of the partners and *b* is a reduction of the equivalence scale. We set $b=1$ since our focus is on individuals and we do not apply the type of equivalence scale common in the literature on income inequality. Kuypers and Marx (2018) examine the sensitivity of annuitized wealth with respect to *b* and find that setting $b=1$ in contrast to values below 1 tends to increase annuitized wealth of older generations relative to annuitized wealth of younger generations.

Since we only observe age of the “household head,” i.e. typically the male spouse, we subtract two years from this number to get the relevant age for both spouses. This corresponds to the rounded average age gap of married couples in Lucerne in 2015.^11^ Note that the gap is not age-invariant. The mean age gap among couples with a husband aged between 20 and 34 is 0.9 years and increases to 3.4 years among couples with a husband aged 65 and older. By assuming a constant age gap, we tend to underestimate the length of annuities and, thus, overestimate the annuitized wealth of the elderly. However, we provide robustness checks in Table 9 in Appendix B showing that the empirical distribution of annuitized wealth as well as the one of joint income-wealth hardly change if we assume age-group varying gaps between spouses.

Like other variables, we split annuitized wealth in half between partners of married or registered couples. For single households, we use the age recorded in our tax data and the gender-specific life expectancy from the mortality tables to measure *n*.

As the relationship between wealth and income changes over the life cycle, we will show how the joint distributions differ across age groups. Again, we subtract two years from the age of the male household head to impute age of married women. The youngest age group in our data consists of 763,000 individuals that are between 20 and 34 years old. Note that individuals in that age group have to file their taxes separately even if they still share a household with their parents, which we do not observe. We obtain 901,000 individuals that are 35–49 years old, 770,000 that are 50-64 years old and, finally, 682,000 that are 65 or older.^12^ Note that the age distribution in Lucerne is similar to the one of entire Switzerland.^13^ Differences in distributional patterns between the 1.56 million men and 1.65 million women in our individual data are not a focus of this paper as gender differences may, to a large extent, be driven by our assumption of equal division of resources within couples.

Our data do not suffer from changes in the tax base that often affect the assessment of factor incomes (Bartels and Jenderny 2015). Another broadly discussed topic is the extent to which personal income tax returns capture “business income,” which may also change over time (Alstadsæter et al. 2016). While it is not entirely clear to what extent income from self-employment includes business income, wealth stocks in our data include self-reported business assets.

## Joint Distribution of Labor Income and Annuitized Wealth

### The Marginal Distributions

Labor income is more important than annuitized wealth for most individuals in Lucerne. Average labor income amounts to 46,516 CHF, whereas average annuitized wealth in our baseline definition with heterogeneous returns is 20,786 CHF (see Table 4 in Appendix).

**Fig. 1:**
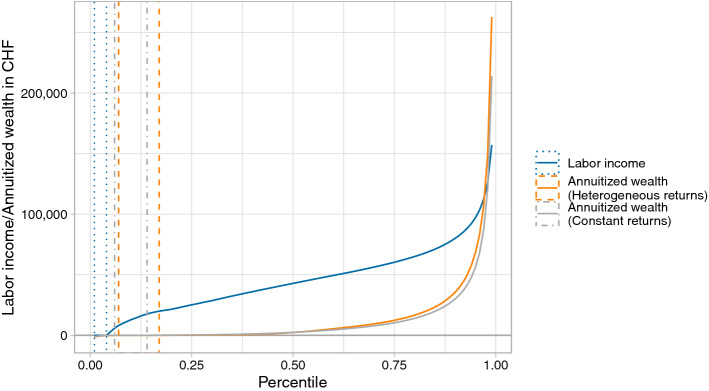
Percentiles of labor income, annuitized wealth with heterogeneous returns, and annuitized wealth with constant returns, respectively, in Lucerne, 2005-2015. Dotted and dashed lines delimit the ranges of percentiles with zero labor income or annuitized wealth

The distribution of annuitized wealth is heavily skewed and much more unequal than the distribution of labor income. For the large majority, annuitized wealth is of secondary importance if not negligible. 17% have either no or negative annuitized wealth and median annuitized wealth is only 2310 CHF. Those at the top of the distribution, on the other hand, have very high annuitized wealth. For instance, the top percentile is 262,823 CHF and is—like all other quantiles above the 98th percentile—higher than the same percentile in the labor income distribution (see Fig. 1).

Figure 1 also shows that allowing for heterogeneous returns matters. There is a significant and throughout the distribution growing difference between annuitized wealth with heterogeneous returns and annuitized wealth with constant returns. Annuitized wealth measured with constant returns is higher below the median but lower at the upper end of the distribution. The 99th percentile of annuitized wealth with heterogeneous is 48,661 CHF (22.7%) higher returns than the same perecentile of annuitized wealth with constant returns.

We also observe substantial differences if we do not correct for the undervaluation of declared real estate wealth by inflating real estate wealth by 150%. Figure 10 in Appendix D visualizes the main differences: First, there are more individuals with negative annuitized wealth, because mortgage debt is not undervalued in tax declarations. Second, since their net wealth more likely consists of real estate wealth, the upper-middle class becomes relatively poorer. Annuitized wealth without the real estate correction is hence on average lower and more unequally distributed than annuitized in our baseline definition (see also Table 9).

Looking at the entire population obscures important differences across age groups. Not surprisingly, labor income increases with age until retirement. However, it increases not uniformly. Labor income is more unequal among individuals aged 50 to 64 years than among individuals from younger cohorts. Finally, labor income decreases with retirement. The average individual 65 and older receives less labor income than individuals just before retirement.

Lower earnings of the elderly obviously contribute to this result. The design of the Swiss pension system matters, as well. For many retirees, AHV pay-as-you-go pensions are the most important source of labor income. Capital-backed pensions, which are mandatory for employees with yearly earnings above 21,150 CHF in 2015, are usually lower. This is not only due to pensions savings that do not match AHV entitlements but also due to the choice of about a third of all newly retired pensioners who disburse their pension savings (Bundesamt für Statistik 2021a). This is relevant in our context as savings converted into life-long monthly pensions are counted as (labor) income flows in our data while disbursed savings appear as (annuitized) wealth stocks.

Pension capital, life-time savings in general, and bequests that benefit individuals typically towards the end of their working life or even in retirement (Stutz et al. 2007; Jann and Fluder 2015; Martínez 2020), all contribute to wealth holdings that sharply increase with age.^14^ The steep age-wealth profiles are shown in Table 5 in Appendix. For annuitized wealth, the differences between age groups are even more pronounced as annuitized wealth does not only depend positively on wealth but also negatively on the remaining life span, i.e. the duration of the annuity (see Table 6 in Appendix). The elderly have on average higher annuitized wealth because, among them, a given wealth stock does not have to guarantee consumption for as long as among younger cohorts.

While individuals above age 65, on average, have high annuitized wealth, there also is a lot of wealth inequality among individuals in that age group. The ten percent with the lowest amount of annuitized wealth among those aged 65 or older only have 135 CHF or less. The amounts at the top of the distribution (e.g. the ninth decile is 97,257 CHF and the 99th percentile is 647,473 CHF) are, in comparison, substantial. Labor income, on the other hand, is more evenly distributed. The first and the ninth deciles of the labor income distribution are relatively close at 17,178 CHF and 53,662 CHF, respectively. For the majority of individuals over 65, labor income is a more important financial resource than annuitized wealth.

**Fig. 2:**
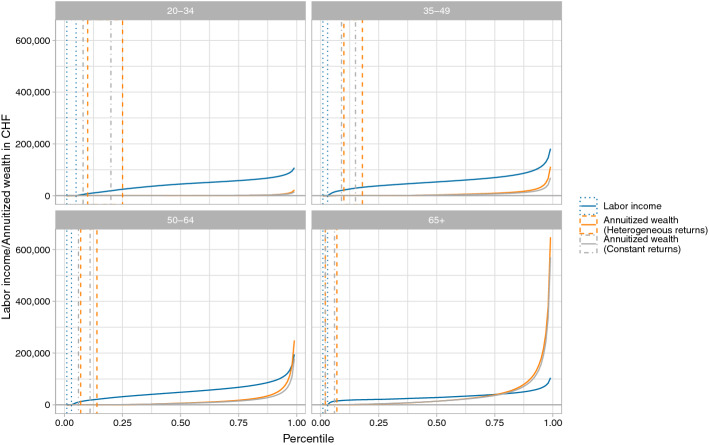
Percentiles of labor income, annuitized wealth with heterogenous returns, and annuitized wealth with constant returns, respectively, by age group, in Lucerne, 2005–2015. Dotted and dashed lines delimit the ranges of percentiles with zero labor income or annuitized wealth

Inequality of annuitized wealth is not limited to the elderly. It is even higher within the youngest age group with a Gini coefficient of 0.899 as wealth is largely irrelevant for most young individuals with the exception of a wealthy few. Wealth inequality decreases substantially over the working life. However, only the top percentile of annuitized wealth among individuals between 50–64 is larger than the corresponding percentile of labor income. For many shortly before retirement, annuitized wealth plays only a secondary role.

Annuitized wealth can be negative if annuitized assets are lower than annuitized debt. The shaded area in Fig. 2 indicate the percentile range with zero labor income or annuitized wealth. To the left of this area, we display the percentile range comprising individuals with negative annuitized wealth. This negative part varies significantly across age groups. Negative annuitized wealth is most prevalent among the 35-to-49-year-old, an age when people start buying houses. Negative annuitized wealth decreases as individuals get older. However, even 1.4% of individuals 65 and older have negative annuitized wealth.

Finally, note that analyzing single sample years instead of the pooled sample, as we have done so far, leads to similar findings. Apart from trend growth in mean labor income and annuitized wealth, we observe relatively stable distributions between 2005 and 2015. Labor income inequality remains virtually unchanged (see Fig. 7 in Appendix B). Annuitized wealth concentration decreased slightly after the Great Recession.

### Dependence Between Labor Income and Annuitized Wealth

The high degree of annuitized wealth inequality suggests that overall economic inequality in the canton of Lucerne will likely be greater if we use a measure that accounts for both labor income and annuitized wealth, such as joint income-wealth, compared to a measure based only on current income. The extent of joint income-wealth inequality, however, depends not only on the marginal distributions but also on the dependence of labor income and annuitized wealth. So, do the labor income rich also have high annuitized wealth?

**Fig. 3:**
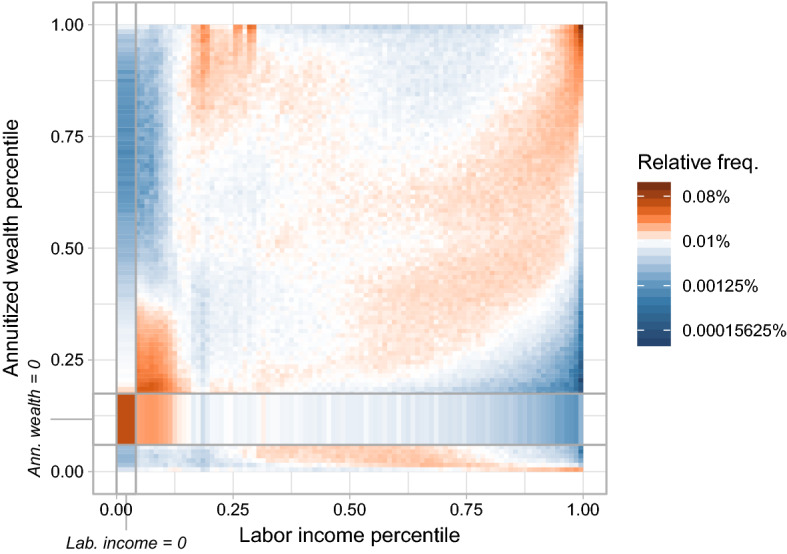
Association matrix between labor income and annuitized wealth (with heterogeneous returns) percentile ranks. Every cell gives the empirical probability of a percentile rank combination, i.e. ${\hat{c}}_{u^\ell _x,u^\ell _y}=\frac{1}{N}\sum _{i=1}^N\mathbbm {1}_{\{u^\ell _x-0.01<{\hat{u}}_{x,i}\le u^\ell _x,u^\ell _y-0.01<{\hat{u}}_{y,i}\le u^\ell _y\}}$ with percentile ranks $u^\ell _{x},u^\ell _{y} \in \{0.01,0.02,\ldots ,1\}$ and empirical quantile ranks ${\hat{u}}_{x},{\hat{u}}_{y}$. Probability mass is distributed uniformly over percentile ranks covered by a mass point where percentiles are not unique like at zero labor income or zero annuitized wealth. Bold lines separate percentile range with zero labor income and annuitized wealth, respectively. The association matrix between current income (including disbursed capital income) and net wealth is shown in Fig. 13 in Appendix E

**Table 1:** Association between Labor Income & Annuitized Wealth by Age Group

	All	20–34	35–49	50–64	65+
*Spearman’s Rank Correlation*					
All Observations	0.218	0.337	0.307	0.294	0.270
Ann. Wealth $>0$	0.155	0.377	0.246	0.229	0.198
Ann. Wealth $<0$	− 0.169	− 0.173	− 0.170	− 0.163	− 0.150
*Share of Jointly Affluent*					
% of Top Lab. Income 1% in Top Ann. Wealth 1%	12.5	10.7	19.7	21.4	12.6
% of Top Lab. Income 10% in Top Ann. Wealth 10%	19.9	27.3	29.7	30.3	24.9

Figure 3 shows the association matrix between percentile ranks of labor income and annuitized wealth with heterogeneous returns. As a discrete approximation to the empirical copula density, every cell of the matrix represents the empirical probability of a percentile combination.

The dependence between labor income and annuitized wealth is positive for individuals with positive annuitized wealth. The degree of dependence is rather weak, though. Spearman’s rank correlation coefficient attains merely 0.134 (see Table 1 for this and other association measures). In particular, the association is not very pronounced for ranks in the middle of the distribution. The convex shaped region of high probability mass in the right half of the association matrix points to an asymmetric association between labor income and annuitized wealth ranks. Individuals in higher labor income percentiles tend to rank in similar but slightly lower annuitized wealth percentiles. This asymmetry reflects the fact that savings out of high labor income are not the only pathway to high annuitized wealth. Persistently high capital income, bequests, or disbursed capital from pension plans are, potentially, an even more important source of high annuitized wealth.

The dependence between labor income and annuitized wealth becomes stronger in the upper tail. High labor income ranks tend to be associated with high annuitized wealth ranks. An individual in the top 1% (10%) of labor income has a 12.5% (19.9%) chance to be part of the top 1% (10%) of annuitized wealth. The dependence is also significant in the lower tail of the distribution for positive annuitized wealth. Figure 3 shows a large share of people with low labor income and low annuitized wealth. 86% of the bottom 10% of the labor income distribution have annuitized wealth below the median. With 3% of all observations, there is also a large number of individuals who have neither labor income nor annuitized wealth such as students or recipients of social assistance, which is tax exempt and, thus, does not appear as income in our data.

The relationship between labor income and annuitized wealth switches signs as we move from positive to negative annuitized wealth. Individuals with negative wealth tend to have high labor income and the more negative their wealth, the higher the incomes are in our data. We observe hardly any combinations of negative annuitized wealth with low labor incomes.

There are two potential economic explanations for the negative association between debt and high labor incomes. First, high-income earners might be more creditworthy or more able to advance the required minimum equity to acquire real estate mortgages than individuals with low labor income. A second potential explanation are self-employed who use debt to finance investments that generate high self-employed income. Krapf’s (2018) finding that individuals with negative net wealth in one year tend to realize high incomes in the following years provides some support for the latter explanation.

Underreported wealth and real estate that remains undervalued even after our correction may, to a certain extent, be responsible for the large number of individuals with negative net wealth in our data. However, they do likely not explain why the association between labor income and wealth switches signs at zero net wealth. As argued in Krapf (2018), tax evasion would, if anything, provide a rationale for a discontinuity at the taxable wealth threshold and not at zero net wealth.

**Fig. 4:**
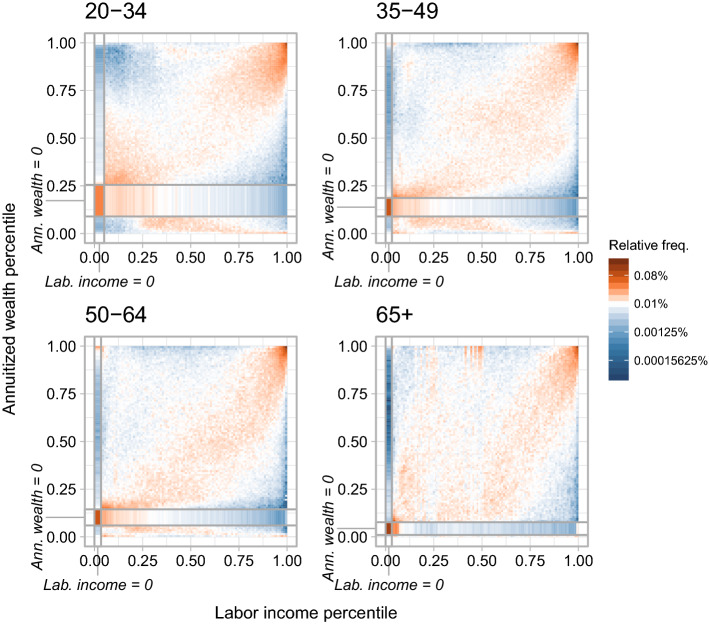
Association matrices between labor income and annuitized wealth (with heterogeneous returns) percentile ranks by age group. Every cell gives the empirical probability of a percentile rank combination, i.e. ${\hat{c}}_{u^\ell _x,u^\ell _y}=\frac{1}{N}\sum _{i=1}^N\mathbbm {1}_{\{u^\ell _x-0.01<{\hat{u}}_{x,i}\le u^\ell _x,u^\ell _y-0.01<{\hat{u}}_{y,i}\le u^\ell _y\}}$ with percentile ranks $u^\ell _{x},u^\ell _{y} \in \{0.01,0.02,\ldots ,1\}$ and empirical quantile ranks ${\hat{u}}_{x},{\hat{u}}_{y}$. Probability mass is distributed uniformly over percentile ranks covered by a mass point where percentiles are not unique like at zero labor income or zero annuitized wealth. Note that percentiles refer to conditional percentiles of respective age groups. Bold lines separate percentile range with zero labor income and annuitized wealth, respectively

“Classical capitalists,” i.e. rentiers with very high annuitized wealth but no labor income are rare. Only 0.006% of all observations are part of the top 1% of the annuitized wealth distribution and do not receive any labor income. There is, however, a significant number of individuals with low labor income within high wealth ranks as indicated by the probability mass in the upper-left part of Fig. 3. One half of the top 1% of the annuitized wealth distribution, for example, have labor income that is below the median. The negative relationship between low labor income and high annuitized wealth is driven by differences across age groups. Many retirees have high wealth but receive labor income only in form of a relatively small public AHV pension (see also Moser 2019), which leads to the high mass in the upper-left corner of Fig. 3.

Consequently, we do not observe high shares of rank combinations between low labor income and high annuitized wealth when we plot the association matrix separately for different age groups in Fig. 4. The dependence between labor income and annuitized wealth for positive wealth exhibits largely symmetric positive patterns in all age groups. We find the highest association as measured by the rank correlation as well as by the share of individuals in the top tail of both distributions among the 50-to-64-years old. For the oldest age group the dependence pattern is still positive but somewhat noisier. One possible explanation is again the system of professional pension savings in Switzerland mentioned above. Individuals who choose to cash-out their pension savings upon retirement have relatively high taxable wealth, whereas individuals who choose to convert their pension savings into a life-long monthly pension have relatively high taxable income.^15^

The sign and the patterns do not fundamentally change if we use alternative specifications of labor income and annuitized wealth or restrict our analysis to a single sample year (see additional association matrices in Appendix E). If we use homogeneous instead of heterogeneous returns we see a slightly weaker assocation between labor income and annuitized wealth for individuals with positive wealth, whereas the negative association among individuals with negative wealth becomes slightly stronger. Without the correction for real estate wealth, the negative association among individuals with negative annuitized wealth becomes stronger.^16^

Overall, we find a positive association between labor income and annuitized wealth, which is slightly attenuated by the negative association between the two financial resources for individuals with negative annuitized wealth and by differences across age groups. We therefore expect that wealth tends to raise inequality not only through its unequal marginal distribution but also through its mostly positive dependence on labor income. We will next inspect the distribution of joint income-wealth and ask how wealth and the dependence structure are shaping it.

## The Distribution of Joint Income-Wealth

Joint income-wealth is on average higher than current income. While the former amounts to 67,302 CHF on average, the latter is 51,470 CHF on average (see Table 5). However, we do not only underestimate the level but also the inequality of financial resources when focusing on current income: The top 1% share of joint income-wealth at 14.5% is almost twice as large as the top 1% share of current income. This raises the question whose financial resources we underestimate if we ignore annuitized wealth.

**Fig. 5:**
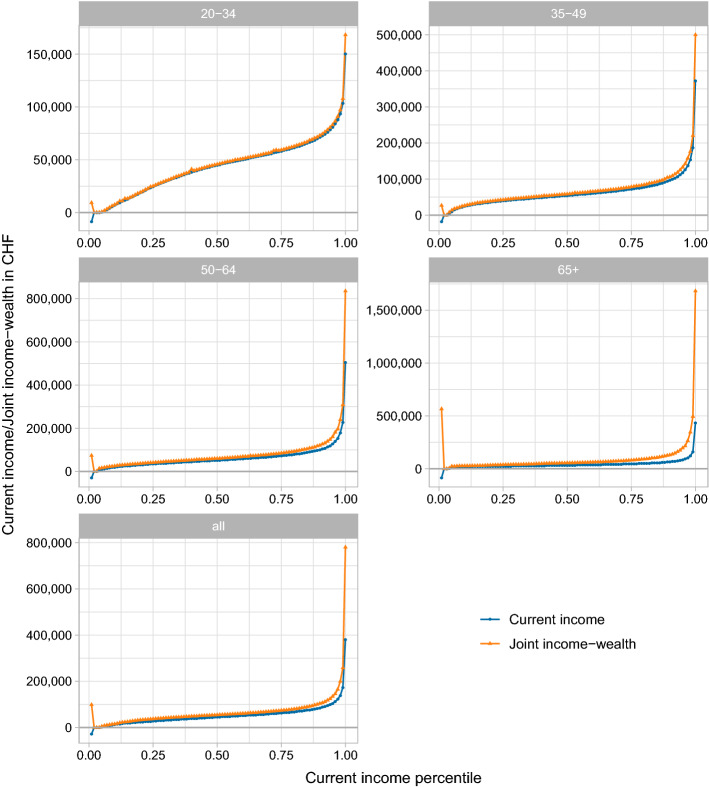
Average joint income-wealth (with heterogeneous returns) and average current income (including capital income) by percentile of the current income distribution and age group

Similar in spirit to Bourguignon’s (2011) “non-anonymous growth incidence curves”, Figure 5 present an answer by plotting mean joint income-wealth and mean current income by percentile of the current income distribution. We use our default version of joint income-wealth with heterogeneous returns. We show patterns for the entire population and the four age groups separately.

Two observations in Fig. 5 are noteworthy. First, wealth is positively correlated with current income. The gap between joint income-wealth and current income is therefore largest at the top of the current income distribution. Average joint income-wealth of the top 1% of the current income distribution, for example, is 399,149 CHF or 85% higher than their mean current income in the same percentile. Further down the distribution, the differences are smaller both in absolute and relative terms. The gap within the 50th percentile of the entire population is 9336 CHF (21% of mean current income) and the gap within the 10th percentile is 2410 CHF (19% of mean current income). The only exception is the first percentile of current income. While individuals in this percentile have on average *negative* current income, they have positive joint income-wealth that is significantly higher than joint income-wealth of other low current income percentiles. A disproportionate share of individuals with high wealth among those with zero or negative labor income accounts for this result.

Second, the gap between joint income-wealth and current income increases with age. For the top 1% among individuals 65 and older, the mean gap is 1,247,120 CHF (286%), which is substantially larger than among younger age groups. Further down the distribution of current income, where the gap between current income and joint income-wealth is less pronounced, this pattern across age groups still holds. In the oldest age group, for example, the gap is 22,345 CHF (68%) at the 50th percentile or 9275 CHF (51%) at the 10th percentile. The gap between the two measures of financial resources among the youngest age group, in contrast, is negligible over large parts of the distribution.

The elderly have, on average, substantially more financial resources than their current income suggests. Mean joint income-wealth within the 50th percentile of the current income distribution of those 65 and older is 55,120 CHF and even slightly higher than mean joint income-wealth within the 50th percentile of the entire population at 54,692 CHF. Among the elderly, however, a larger share of joint income-wealth comes from annuitized wealth than from labor income. However, this does not mean that all elderly have a lot of wealth to increase their consumption possibilities. Wealth is, in fact, distributed very unequally among the elderly.

As a consequence, inequality within the oldest age group is larger if we look at joint income-wealth rather than at current income. The Gini coefficient substantially increases from 0.370 for current income to 0.562 for joint income-wealth (see again Table 5). For younger age groups, the difference in inequality is substantially lower. The Gini coefficients of current income and joint income-wealth among 50-to-64-years-old individuals are 0.370 for current income and 0.422 for joint income-wealth. We thus tend to underestimate inequality in consumption possibilities if we ignore wealth, particularly among the elderly.

Our results are robust to applying alternative definitions of annuitized wealth. Figure 9 in Appendix D compares current income percentiles to respective percentiles of different joint income-wealth definitions. Figure 12 compares percentiles of baseline definition of joint income-wealth to the alternative definitions of joint income-wealth. Table 8 in Appendix B provides additional descriptive statistics for the different definitions.

## Decomposing Joint Income-Wealth Inequality

In this section, we explore how labor income, annuitized wealth, and the dependence among the two underlying factors shape the distribution of joint income-wealth. We build on the copula decomposition of Rothe (2015), which decomposes differences across the distribution of an outcome variable, into the contributions of single explanatory variables and into the contribution of the dependence among those variables.

We use Rothe’s approach to understand how labor income and annuitized wealth translate into joint income-wealth inequality. To this end, we compare the observed distribution of joint income-wealth to the hypothetical scenario of perfect equality where every individual has mean joint income-wealth. We then decompose the observed difference between the observed distribution and the point mass distribution under equality into the contribution of the two marginal distributions and their dependence structure, respectively. Appendix F provides a formal description of the decomposition terms and provides details about their estimation.

To isolate the role of the dependence structure, what we call the “dependence effect”, we compare the actual distribution of joint income-wealth to a counterfactual distribution assuming that labor income and annuitized wealth were independent. We derive this counterfactual by linking the two marginals with the independence copula. The dependence effect tells us by how much of overall inequality is due to annuitized wealth depending on labor income, e.g., individuals with high labor income are more likely to have high annuitized wealth than individuals with low labor income.

We then measure the contribution of labor income by contrasting the counterfactual under independence to a counterfactual where annuitized wealth is distributed as observed but labor income is equally distributed, i.e., everyone had mean labor income. Similarly, the contribution of annuitized wealth corresponds to the difference between the independence counterfactual and a scenario where everybody has mean annuitized wealth.

Finally, we identify an “interaction effect”. This remainder term captures the difference between the counterfactual under independence and the scenario under perfect equality which is not explained by the contributions of the two marginals alone. Even as we deal with independent marginals, the interaction effect is generally not zero. Inequality in the sum of two independent factors usually exceeds the sum of the inequalities in one factor and the mean of the respective other factor (see analytical example in Appendix F).

There are also alternative decomposition methods. The additively separable Theil index or classical Gini decomposition are, however, uninformative about the contribution of the dependence structure. For comparison, we will still provide a classical Gini decomposition and, related, the decomposition of average joint income-wealth into average labor income and average annuitized by percentile in Appendix F.

**Fig. 6:**
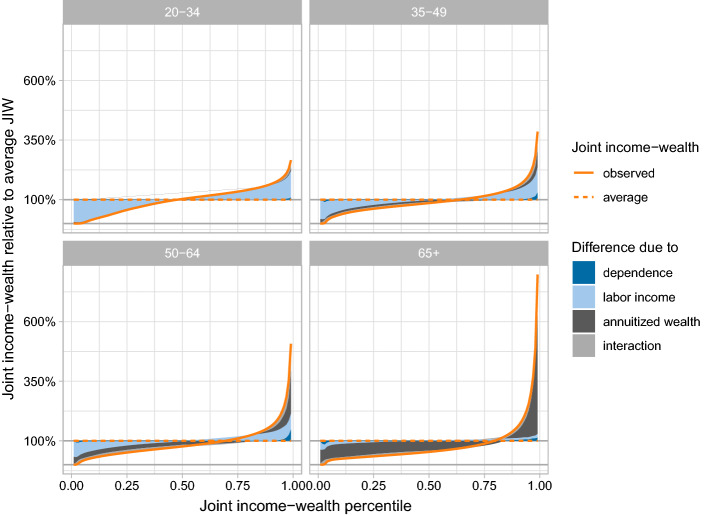
Decomposition of difference between observed joint income-wealth percentiles and percentiles under perfect equality if every individual had mean joint income-wealth, by age group. The shaded areas show the contributions of the distribution of income, the distribution of annuitized wealth, the interaction between the two distributions, and the dependence between the distributions, respectively. See text for discussion and Appendix F for a formal description of the decomposition

**Fig. 7:**
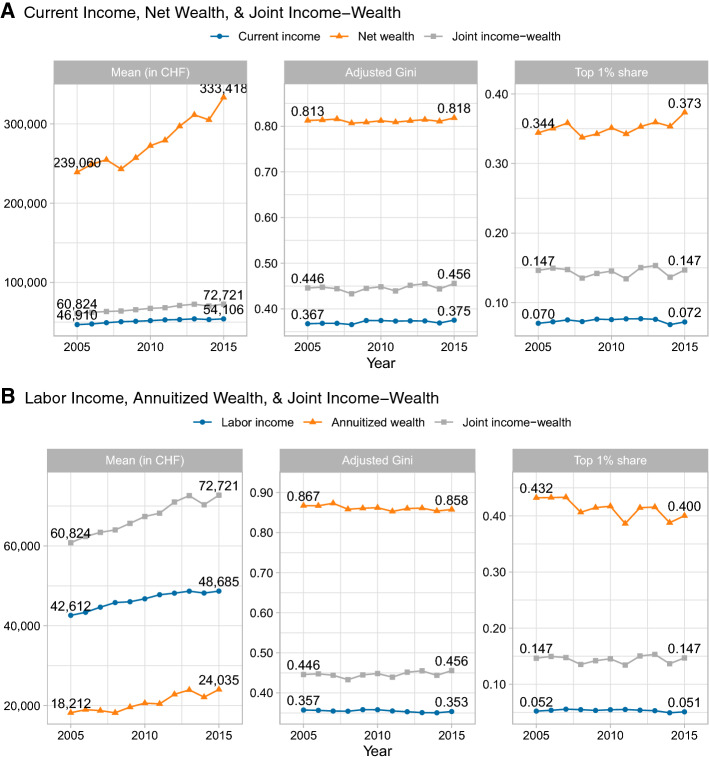
Descriptive statistics of main variables in Lucerne by sample year. Annuitized wealth and joint income-wealth are calculated with heterogeneous returns. Annuitized wealth, joint income-wealth, and net wealth are adjusted for undervaluation of declared real estate wealth. Gini coefficient is adjusted for negative values and normalized to $\left[ 0,1\right]$-range (see Chen et al. 1982)

**Fig. 9:**
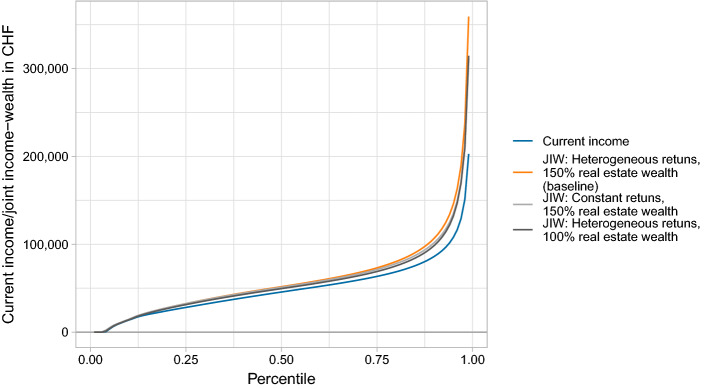
Percentiles of current income and joint income-wealth (JIW) based on different definitions of annuitized wealth in Lucerne, 2005–2015. We use annuitized wealth with heterogeneous returns and declared real estate wealth inflated by 150% as baseline definition

**Fig. 10:**
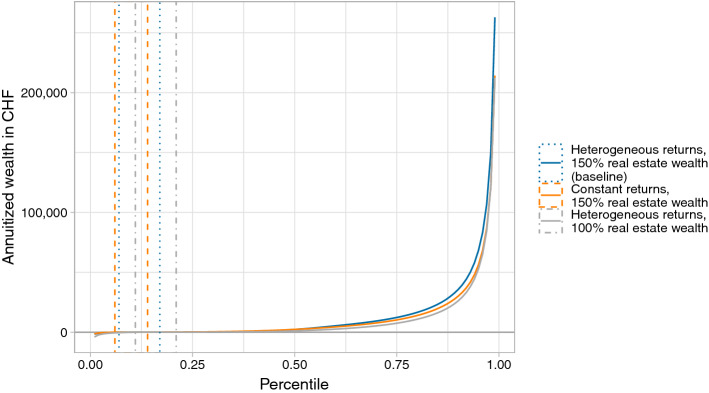
Percentiles of annuitized wealth by alternative definition in Lucerne, 2005-2015. We use annuitized wealth with heterogeneous returns and declared real estate wealth inflated by 150% as baseline definition. Dotted and dashed lines delimit the ranges of percentiles with zero annuitized wealth

**Fig. 12:**
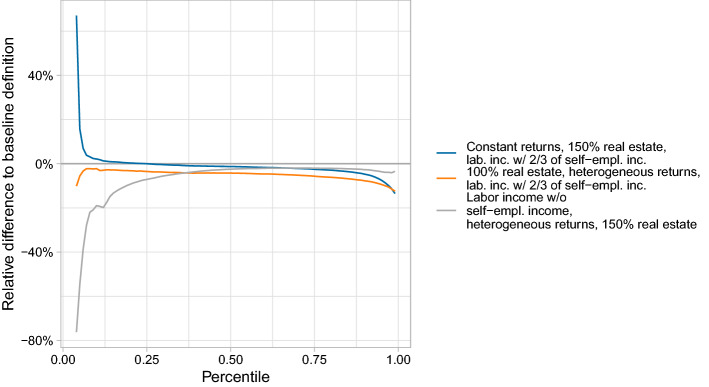
Relative difference between alternative joint income-wealth definitions and baseline definition, by percentile, 2005–2015. Baseline definition—as shown in 8—is defined as the sum of labor income (including 2/3 of self-employed income) and annuitized wealth with heterogeneous returns and real estate wealth inflated by 150%. Alternative definitions either exclude self-employment income from labor income, calculate with constant returns, or do not inflate declared real estate wealth. A negative value indicates that a given percentile of alternative definition is lower than the baseline definition

Figure 6 presents the results of the copula decomposition for the four age groups. The solid orange lines display observed percentiles of joint income-wealth by age group relative to the age group-specific mean. The dashed orange lines, on the other hand, correspond to the equality scenario where every individual has mean joint income-wealth. The shaded areas represent the decomposition terms and show by how much the observed percentiles differ from percentiles under equality due to the labor income distribution, the annuitized wealth distribution, and the dependence among the two distributions.

The dependence structure explains only a minor part of joint income-wealth inequality (see dark blue areas). Even if labor income and annuitized wealth were independent, i.e. if all quantile rank combinations were equally likely, inequality would not be much smaller. The dependence is most relevant for those 50 to 64 years old where it contributes positively to joint income-wealth in the upper tail. This reflects the above observation that upper-tail dependence between the two distributions is most pronounced for individuals shortly before retirement. Even within this group, its contribution is small, though. For instance, the top 1% share of joint income-wealth would only be 0.9 percentage points (or 4%) smaller if labor income and annuitized wealth were independent (see Table 2).

**Table 2:** Decomposition of Top 1% Share Difference* of Joint Income-Wealth

	20–34	35–49	50–64	65+	All
Dependence	0.002	0.008	0.009	0.006	0.007
Income	0.013	0.012	0.008	0.002	0.005
Annuitized Wealth	0.008	0.033	0.072	0.192	0.098
Interaction	0.009	0.020	0.026	0.016	0.025
Top 1% Share Difference*	0.032	0.073	0.115	0.216	0.135

*Difference to top 1% share under equality: $\Delta ={\hat{S}}^{\text {Top 1}\%}-0.01$

The two marginal distributions are more relevant than the dependence structure. Labor income essentially shapes the distribution of joint income-wealth for the two younger age groups (light blue area). Annuitized wealth within those age groups is generally low (dark grey area). High inequality among individuals 65 and older, in contrast, is mostly driven by annuitized wealth. Even among individuals aged 50 to 64, labor income shapes the distribution of joint income-wealth over large parts. Annuitized wealth contributes to the concentration of joint income-wealth at the top in this age group. For illustration, the top 1% share of joint income-wealth of those 50 to 64 years old would be 32% smaller if annuitized wealth was distributed equally.

Finally, we see that the two marginal distributions interact. Even if they were independent, inequality of joint income-wealth measured by quantile ratios, top shares, or the Gini coefficient would not just be the sum of inequality of labor income shifted by average annuitized wealth, on the one hand, and of the inequality of annuitized wealth shifted by average labor income, on the other hand. As the “interaction effect” shows (light grey area), it would be higher in all age groups. This highlights again the importance of the two marginal distributions in shaping inequality.

## Conclusion

In this paper, we apply methods from several recent literatures to assess joint income-wealth inequality using tax data from the Swiss canton of Lucerne. To improve measures of consumption possibilities related to wealth, we introduce heterogeneous returns (Fagereng et al. 2020) into the estimation of annuitized wealth (Weisbrod and Hansen 1968). The size and quality of our data allow us to use non-parametric techniques in the spirit of Aaberge et al. (2018) or Chetty et al. (2017) in our estimation of joint distributions. Finally, we use Rothe﻿’s (2015) method to decompose joint income-wealth into four contributing factors.

Inequality in the Swiss canton of Lucerne increases if we base our assessment on joint income-wealth rather than current income. The marginal distribution of annuitized wealth being more unequal than the labor income distribution is the most important factor explaining this increase in inequality. Positive dependence between labor income and annuitized wealth contributes primarily to inequality in the upper tail of the distribution. Labor income and annuitized wealth are, however, negatively associated for negative net annuitized wealth. Annuitized wealth accounts for a large share of consumption possibilities among the elderly, who tend to have high wealth-to-income ratios. But even among the elderly, annuitized wealth is highly concentrated at the top of the distribution.

Our analysis suggests that the determinants of inequality in consumption possibilities differ across age groups. While labor earnings shape the distribution of joint income-wealth among the young, annuitized wealth and capital income play a dominant role among older generations. The association between labor earnings and annuitized wealth, however, does not appear to be a crucial driver of overall inequality. Our findings imply that examinations of tax progressivity and redistribution in Switzerland need to account for both income and wealth taxes.

## Figures and Tables

**Fig. 8:**
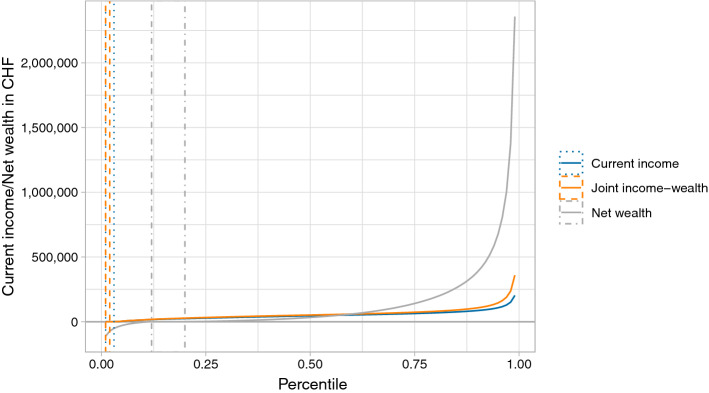
Percentiles of current income, joint income-wealth with heterogeneous returns and net wealth in Lucerne, 2005–2015. Dotted and dashed lines delimit the ranges of percentiles with zero current income, joint income-wealth or net wealth

**Fig. 11:**
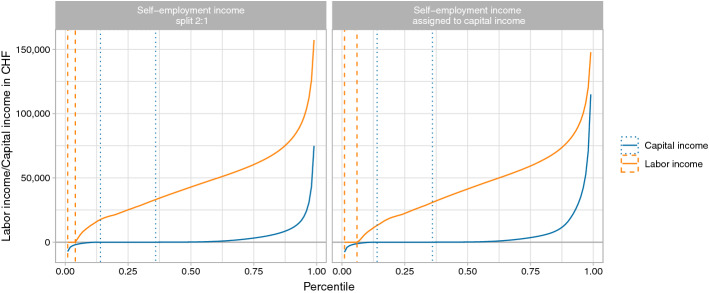
Percentiles of labor income and capital income in Lucerne, 2005-2015. The plot shows two different income definitions. The first attributes two third of self-employment income to labor income and a third to capital income. We apply this definition for all other capital income statistics (see Sect. 3). The second assigns all self-employment income to capital income. Dotted and dashed lines delimit the ranges of percentiles with zero labor income or capital income

**Fig. 13:**
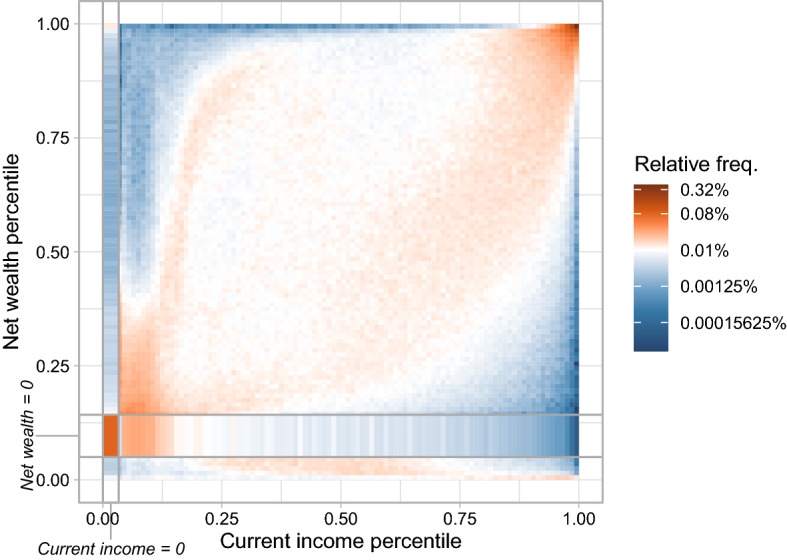
Association matrix between current income and net wealth percentile ranks. Every cell gives the empirical probability of a percentile rank combination, i.e. ${\hat{c}}_{u^\ell _x,u^\ell _y}=\frac{1}{N}\sum _{i=1}^N\mathbbm {1}_{\{u^\ell _x-0.01<{\hat{u}}_{x,i}\le u^\ell _x,u^\ell _y-0.01<{\hat{u}}_{y,i}\le u^\ell _y\}}$ with percentile ranks $u^\ell _{x},u^\ell _{y} \in \{0.01,0.02,\ldots ,1\}$ and empirical quantile ranks ${\hat{u}}_{x},{\hat{u}}_{y}$. Probability mass is distributed uniformly over percentile ranks covered by a mass point where percentiles are not unique like at zero current income or zero net wealth. Bold lines separate percentile range with zero current income and net wealth, respectively

**Fig. 14:**
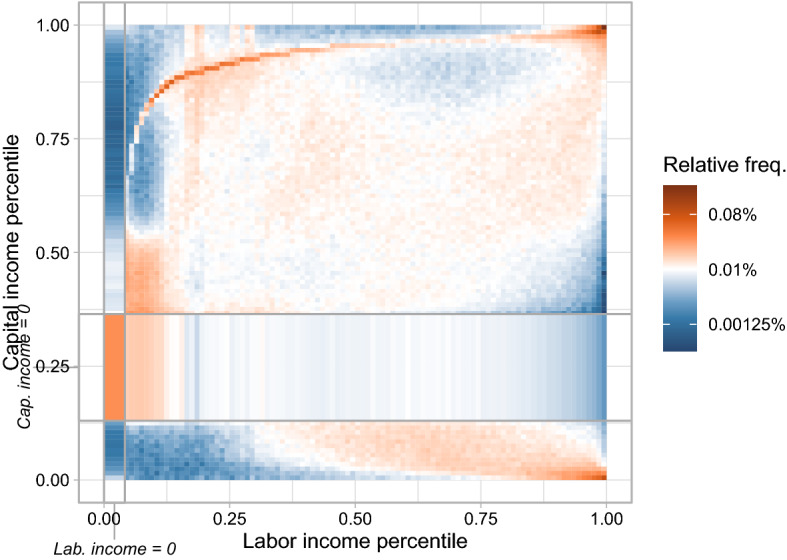
Association matrix between labor and capital income percentile ranks. Two thirds of self-employment income assigned to labor income and the remaining third to capital income. We apply this definition for all other statistics of labor and capital income (see Sect. 3). Every cell gives the empirical probability of a percentile rank combination, i.e. ${\hat{c}}_{u^\ell _x,u^\ell _y}=\frac{1}{N}\sum _{i=1}^N\mathbbm {1}_{\{u^\ell _x-0.01<{\hat{u}}_{x,i}\le u^\ell _x,u^\ell _y-0.01<{\hat{u}}_{y,i}\le u^\ell _y\}}$ with percentile ranks $u^\ell _{x},u^\ell _{y} \in \{0.01,0.02,\ldots ,1\}$ and empirical quantile ranks ${\hat{u}}_{x},{\hat{u}}_{y}$. Probability mass is distributed uniformly over percentile ranks covered by a mass point where percentiles are not unique like at zero labor income or zero capital income. Bold lines separate percentile range with zero labor income and capital income, respectively

**Fig. 15:**
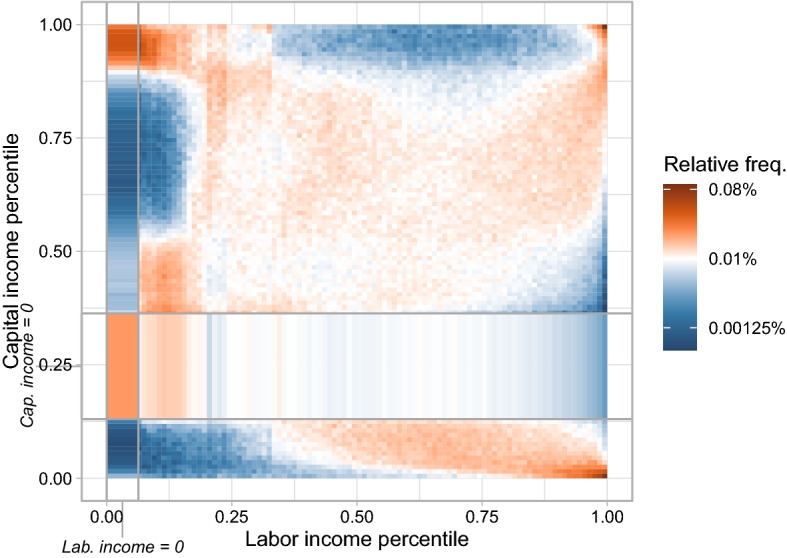
Association matrix between labor and capital income percentile ranks. Self-employment income entirely assigned to capital income. Every cell gives the empirical probability of a percentile rank combination, i.e. ${\hat{c}}_{u^\ell _x,u^\ell _y}=\frac{1}{N}\sum _{i=1}^N\mathbbm {1}_{\{u^\ell _x-0.01<{\hat{u}}_{x,i}\le u^\ell _x,u^\ell _y-0.01<{\hat{u}}_{y,i}\le u^\ell _y\}}$ with percentile ranks $u^\ell _{x},u^\ell _{y} \in \{0.01,0.02,\ldots ,1\}$ and empirical quantile ranks ${\hat{u}}_{x},{\hat{u}}_{y}$. Probability mass is distributed uniformly over percentile ranks covered by a mass point where percentiles are not unique like at zero labor income or zero capital income. Bold lines separate percentile range with zero labor income and capital income, respectively

**Fig. 16:**
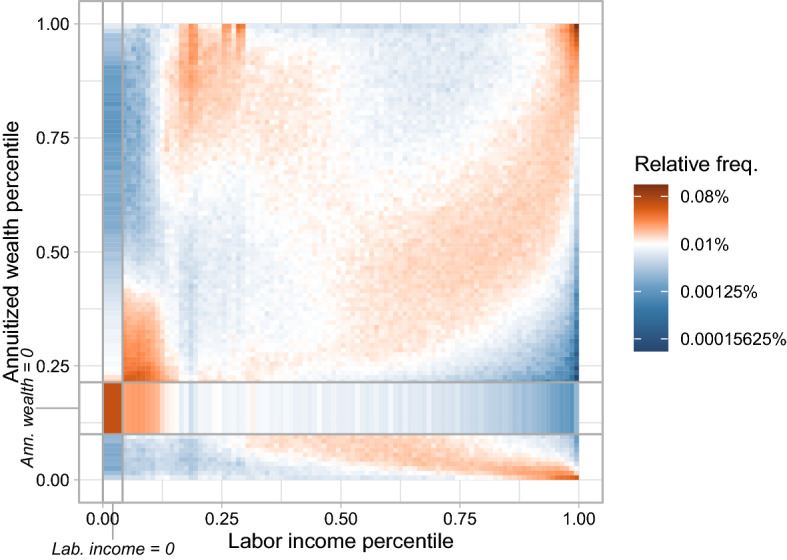
Association matrix between percentile ranks of labor income and annuitized wealth without real estate inflation but with heterogeneous returns. Every cell gives the empirical probability of a percentile rank combination, i.e. ${\hat{c}}_{u^\ell _x,u^\ell _y}=\frac{1}{N}\sum _{i=1}^N\mathbbm {1}_{\{u^\ell _x-0.01<{\hat{u}}_{x,i}\le u^\ell _x,u^\ell _y-0.01<{\hat{u}}_{y,i}\le u^\ell _y\}}$ with percentile ranks $u^\ell _{x},u^\ell _{y} \in \{0.01,0.02,\ldots ,1\}$ and empirical quantile ranks ${\hat{u}}_{x},{\hat{u}}_{y}$. Probability mass is distributed uniformly over percentile ranks covered by a mass point where percentiles are not unique like at zero labor income or zero annuitized wealth. Bold lines separate percentile range with zero labor income and annuitized wealth, respectively

**Fig. 17:**
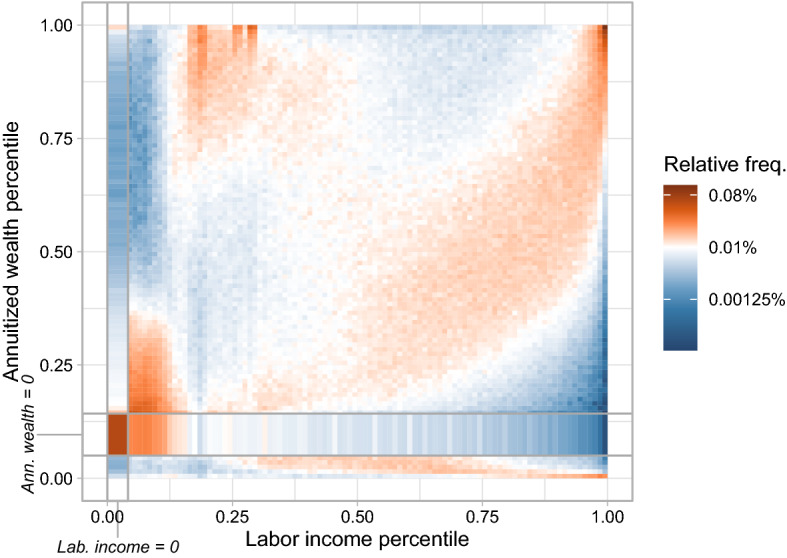
Association matrix between percentile ranks of labor income and annuitized wealth with constant returns. Every cell gives the empirical probability of a percentile rank combination, i.e. ${\hat{c}}_{u^\ell _x,u^\ell _y}=\frac{1}{N}\sum _{i=1}^N\mathbbm {1}_{\{u^\ell _x-0.01<{\hat{u}}_{x,i}\le u^\ell _x,u^\ell _y-0.01<{\hat{u}}_{y,i}\le u^\ell _y\}}$ with percentile ranks $u^\ell _{x},u^\ell _{y} \in \{0.01,0.02,\ldots ,1\}$ and empirical quantile ranks ${\hat{u}}_{x},{\hat{u}}_{y}$. Probability mass is distributed uniformly over percentile ranks covered by a mass point where percentiles are not unique like at zero labor income or zero annuitized wealth. Bold lines separate percentile range with zero labor income and annuitized wealth, respectively

**Fig. 18:**
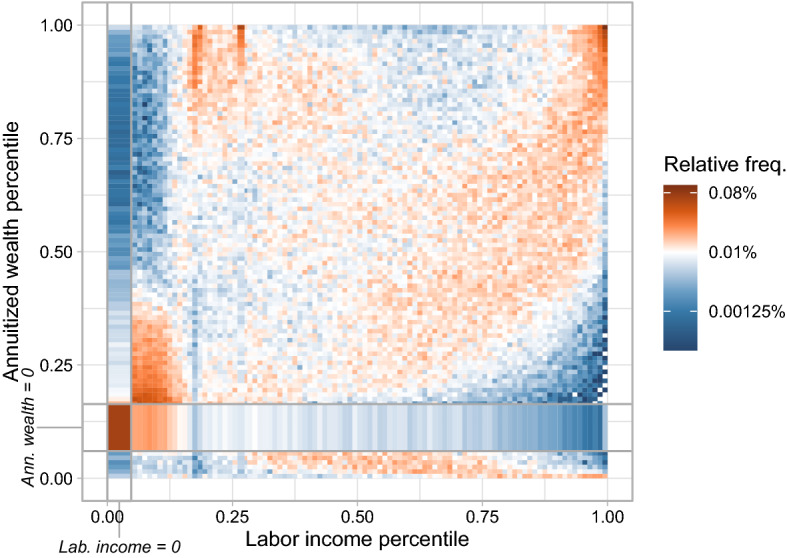
Association matrix between percentile ranks of labor income and annuitized wealth with heterogeneous returns, only with observations from 2015. Every cell gives the empirical probability of a percentile rank combination, i.e. ${\hat{c}}_{u^\ell _x,u^\ell _y}=\frac{1}{N}\sum _{i=1}^N\mathbbm {1}_{\{u^\ell _x-0.01<{\hat{u}}_{x,i}\le u^\ell _x,u^\ell _y-0.01<{\hat{u}}_{y,i}\le u^\ell _y\}}$ with percentile ranks $u^\ell _{x},u^\ell _{y} \in \{0.01,0.02,\ldots ,1\}$ and empirical quantile ranks ${\hat{u}}_{x},{\hat{u}}_{y}$. Probability mass is distributed uniformly over percentile ranks covered by a mass point where percentiles are not unique like at zero labor income or zero annuitized wealth. Bold lines separate percentile range with zero labor income and annuitized wealth, respectively

**Fig. 19:**
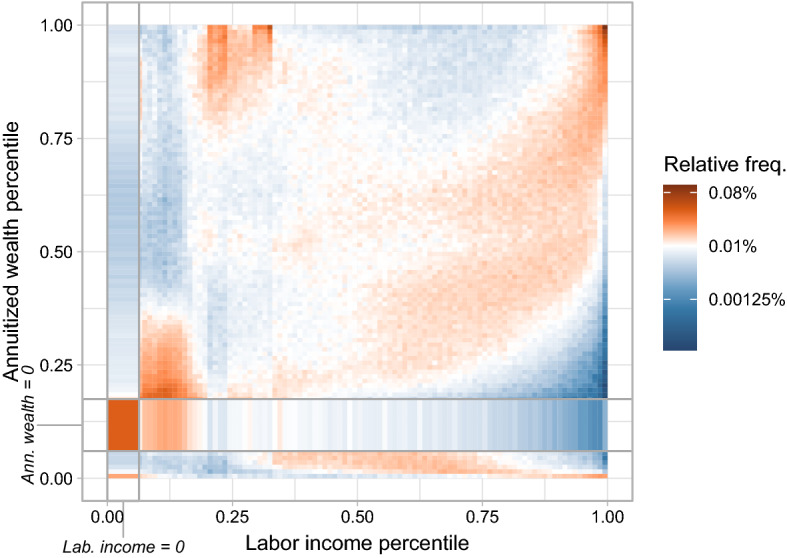
Association matrix between percentile ranks of labor income without self-employment income and annuitized wealth with heterogeneous returns. Every cell gives the empirical probability of a percentile rank combination, i.e. ${\hat{c}}_{u^\ell _x,u^\ell _y}=\frac{1}{N}\sum _{i=1}^N\mathbbm {1}_{\{u^\ell _x-0.01<{\hat{u}}_{x,i}\le u^\ell _x,u^\ell _y-0.01<{\hat{u}}_{y,i}\le u^\ell _y\}}$ with percentile ranks $u^\ell _{x},u^\ell _{y} \in \{0.01,0.02,\ldots ,1\}$ and empirical quantile ranks ${\hat{u}}_{x},{\hat{u}}_{y}$. Probability mass is distributed uniformly over percentile ranks covered by a mass point where percentiles are not unique like at zero labor income or zero annuitized wealth. Bold lines separate percentile range with zero labor income and annuitized wealth, respectively

**Fig. 20:**
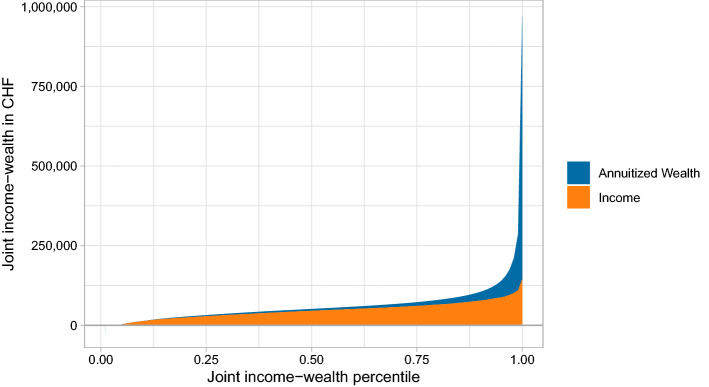
Average income and annuitized wealth (with heterogeneous returns) by joint income-wealth percentile

**Fig. 21:**
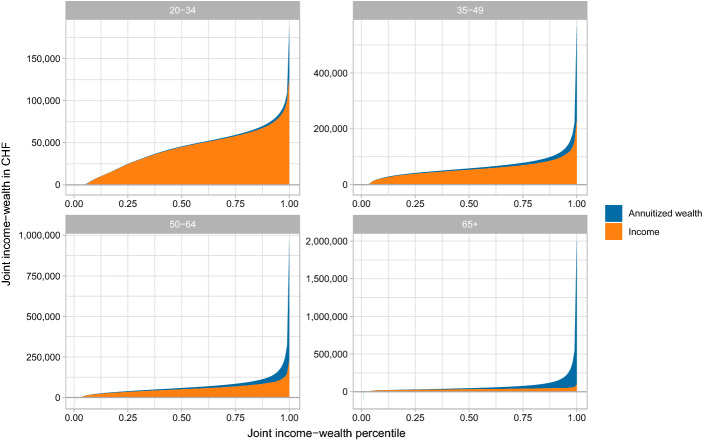
Average labor income and annuitized wealth (with heterogeneous returns) by joint income-wealth percentile by age group

## Data Availability

The data used in this paper covers declared income and wealth of the universe of tax payers in the canton of Lucerne. On submitting a data application with a research proposal, Matthias Krapf was granted access to the microdata by the Statistical Office of Lucerne (LUSTAT). Responsible for our project at LUTSTAT was Mr. Roberto Frisullo. Due to the confidential nature of the data, we cannot grant access to the microdata. However, we provide all code of our empirical analysis as well as the aggregate data required to produce all plots and tables at https://drive.switch.ch/index.php/s/yyNDTy1PlScV96K. At the same location, the file “LUSTAT_variable_list_20180504.xlsx”﻿ lists all variables provided by LUSTAT. We analyzed the data using Stata (StataCorp 2021) and R (R Core Team 2021). The following four scripts need to be run to replicate the results: A - Prepare data Wealth-Income LU WI_LU.do: This Stata do-file constructs all variables from raw tax data. It also computes the frequencies of the joint distributions and estimates correlation coefficients. B - Estimate summary stats.R: This R script computes the descriptive statistics on the tax data. The script loads user-written estimation functions saved in the folder “stat functions”﻿. C - Estimate copula and Gini decomposition.R performs the copula decomposition as well as the Gini coefficient decomposition. The decomposition functions are stored in the folder “stat functions”﻿, too. The R script D - Create plots and tables.R produces all plots and tables shown in this paper. The script calls several procedures saved in the folder “plot functions”﻿.

## A Additional Information on Income Measures

### Appendix A.A Measures Based on Disbursed Taxable Capital Income

In the paper by Atkinson and Lakner (2017) that we rely upon in Sect. 3, they suggest alternative measures of labor and capital income to account for the dependence between capital and labor income induced by the split of income from self-employment. These follow the same methodology, but assign all income from self-employment to capital income and none to labor income. Atkinson and Lakner’s methods lead to higher measures of housing income and lower measures of financial income if applied to our data than to data from other countries. Income from housing wealth includes imputed rents for people who live in homes they own themselves, a feature that is, to our knowledge, unique to Switzerland. Our data do not allow us to distinguish between imputed rents and actual income flows if the property is rented to others. These measures of capital income, hence, consist of more property income than would be the case with similar data from other countries because of imputed rents. Additionally, capital income includes neither accrued nor realized capital gains, which are not taxed in Switzerland.

### Appendix A.B Measures Based on Annuitized Wealth

Annuitized wealth $Y_a$ overcomes the shortcomings of disbursed taxable capital income mentioned above and is thus better comparable to measures from other countries. Alternatively to different asset types, which we use based on Eq. (3), we could also use the stock of net wealth. Fagereng et al. (2020) additionally provide returns that vary along the distribution of net wealth rather than financial assets and real estate as an alternative. Returns on net wealth are, however, potentially less comparable to returns in Switzerland because of different rates of home ownership, real estate valuation and indebtedness.

As we mention in Sect. 3, we take returns on financial assets $r_f^p$ directly from Fagereng et al. (2020). These can be found in Panel A of Fig. 2 of their paper. We approximate these patterns by a piecewise linear function, where we assume that returns are 0.77% in the first percentile of financial asset holding, decrease linearly from 0.4% to 0 between the second and 18th percentile, remain 0 for the 19th and 20th percentiles, then increase linearly to 0.15% for the 30th percentile, to 0.35% for the 40th percentile, to 0.6% for the 50th percentile, to 0.9% for the 60th percentile, to 1.06% for the 64th percentile, to 1.3% for the 70th percentile, to 1.8% for the 80th percentile, to 2.8% for the 90th percentile, to 4% for the 95th percentile, to 4.9% for the 97th percentile to 6.2% for the 99th percentile and, finally, to 6.4% for financial asset holdings in the 100th percentile.

We approximate real estate returns using the asking price index of Wüest Partner (2021) of central Switzerland. They vary between − 1.12% in 2007 and 10.31% in 2012, with a geometric mean of the years of 3.90% over our sample period between 2005 and 2015. We retrieve data from Schweizerische Nationalbank (2021) to assess interest rates on debt. Fagereng et al. (2020) use a return that is constant over time at 2.88%. Since we do not observe debt composition, we use interest on amounts due from domestic customers, which is available only from 2007 onwards and declined from 4.01% to 2.17% over that period. For the remaining years 2005 and 2006, we use the mean ratio of interest on debt and debt stock observed in our data, which are 3.27% and 3.22%. The interest rates over all eleven years result in a geometric mean of 2.78%.

We retrieved the mortality tables from Kohli (2017) to measure remaining life expectancy. The mean age gap for married couples in Switzerland is 2.4 years and has not changed much over time. A possible extension in future work would be to use income- and wealth-dependent life expectancy rates. As argued by Saez and Zucman (2019), accounting for income-dependent life expectancy rates leads to large variation in estimates of tax revenue from a wealth tax for the United States.

## B Descriptive Statistics of Entire Population

See Tables 3 and 4

**Table 3:** Current Income, Joint Income-Wealth, and Net Wealth

	Current Income	Joint Income-Wealth		Net Wealth
		Heterogeneous Returns	Constant Returns	
% obs. with y $= 0$	3.1	2.7	2.4	8.6
% obs. with y $< 0$	0.2	0.2	0.1	5.7
Adj. Gini*	0.372	0.448	0.427	0.813
Top 1% share	0.074	0.145	0.132	0.353
Top 10% share	0.273	0.359	0.338	0.690
Mean (in CHF)	51,470	67,302	64,060	277,740
10th perc. (in CHF)	13,668	14,272	14,575	0
Median (in CHF)	45,677	51,749	51,074	57,038
90th perc. (in CHF)	86,220	106,973	101,868	524,405
99th perc. (in CHF)	202,873	359,209	310,710	3,000,000

*Gini coefficient adjusted for negative values and normalized to $\left[ 0,1\right]$-range (see Chen et al. 1982)

**Table 4:** Income, Capital Income, and Annuitized Wealth

	Labor Income	Capital Income	Annuitized Wealth	
			Heterogeneous Returns	Constant Returns
% obs. with y $= 0$	4.0	23.3	10.7	8.6
% obs. with y $< 0$	0.1	13.2	6.8	5.7
Adj. Gini*	0.356	0.924	0.862	0.855
Top 1% share	0.053	0.412	0.412	0.416
Top 10% share	0.247	0.843	0.766	0.759
Mean (in CHF)	46,516	4,954	20,786	17,543
10th perc. (in CHF)	12,784	− 288	0	0
Median (in CHF)	42,852	119	2,310	2,392
90th perc. (in CHF)	80,099	10,684	35,645	30,096
99th perc. (in CHF)	157,201	74,876	262,824	214,163

*Gini coefficient adjusted for negative values and normalized to $\left[ 0,1\right]$-range (see Chen et al. 1982)

## C Descriptive Statistics by Age Group

See Tables 5, 6, 7, 8, 9

**Table 5:** Net Wealth, Current Income, and Joint Income-Wealth by Age Group

	20–34	35–49	50–64	65+	All
*Net Wealth*					
% obs. with y $< 0$	7.6	8.3	5.4	1.1	5.7
% obs. with y $= 0$	12.6	6.7	6.2	5.7	8.6
Mean (in CHF)	49,435	208,891	383,362	544,723	277,740
Adj. Gini*	0.805	0.771	0.766	0.760	0.813
Top 1% share	0.304	0.311	0.312	0.329	0.353
*Current Income*					
% obs. with y $< 0$	0.1	0.3	0.3	0.1	0.2
% obs. with y $= 0$	3.6	2.6	2.4	3.0	3.1
Mean (in CHF)	43,271	61,426	61,835	41,930	51,470
Adj. Gini*	0.327	0.314	0.370	0.370	0.372
Top 1% share	0.035	0.061	0.082	0.104	0.074
*Joint Income-Wealth (Heterogeneous Returns)*					
% obs. with y $< 0$	0.2	0.2	0.2	0.1	0.2
% obs. with y $= 0$	3.0	2.4	2.1	2.7	2.7
Mean (in CHF)	44,528	68,205	77,266	88,571	67,302
Adj. Gini*	0.335	0.343	0.422	0.562	0.448
Top 1% share	0.042	0.083	0.125	0.226	0.145
*Joint Income-Wealth (Constant Returns)*					
% obs. with y $< 0$	0.1	0.2	0.2	0.1	0.1
% obs. with y $= 0$	2.8	2.0	1.7	2.4	2.4
Mean (in CHF)	44,182	64,747	71,966	84,185	64,060
Adj. Gini*	0.329	0.323	0.394	0.543	0.427
Top 1% share	0.037	0.068	0.106	0.224	0.132

*Gini coefficient adjusted for negative values and normalized to $\left[ 0,1\right]$-range (see Chen et al. 1982)

**Table 6:** Labor Income, Capital Income, and Annuitized Wealth by Age Group

	20–34	35–49	50–64	65+	All
*Labor Income*					
% obs. with y $< 0$	0.0	0.1	0.2	0.0	0.1
% obs. with y $= 0$	5.0	3.1	3.2	3.2	4.0
Mean (in CHF)	42,680	57,339	54,253	33,192	46,516
Adj. Gini*	0.325	0.303	0.348	0.301	0.356
Top 1% share	0.032	0.049	0.057	0.058	0.053
*Capital Income*					
% obs. with y $< 0$	12.9	20.5	15.1	3.6	13.2
% obs. with y $= 0$	39.2	19.4	16.6	15.3	23.3
Mean (in CHF)	592	4086	7583	8738	4954
Adj. Gini*	0.987	0.932	0.896	0.856	0.924
Top 1% share	0.608	0.383	0.359	0.362	0.412
*Annuitized Wealth (Heterogeneous Returns)*					
% obs. with y $< 0$	9.8	9.5	6.2	1.4	6.8
% obs. with y $= 0$	15.7	9.2	8.3	6.4	10.7
Mean (in CHF)	1848	10,866	23,013	55,379	20,786
Adj. Gini*	0.899	0.801	0.795	0.793	0.862
Top 1% share	0.433	0.343	0.337	0.347	0.412
*Annuitized Wealth (Constant Returns)*					
% obs. with y $< 0$	7.6	8.3	5.4	1.1	5.7
% obs. with y $= 0$	12.6	6.7	6.2	5.7	8.6
Mean (in CHF)	1502	7408	17,713	50,994	17,543
Adj. Gini*	0.808	0.775	0.772	0.781	0.855
Top 1% share	0.306	0.314	0.318	0.351	0.416

*Gini coefficient adjusted for negative values and normalized to $\left[ 0,1\right]$-range (see Chen et al. 1982)

**Table 7:** Alternative definitions of annuitized wealth & labor income by age group

	20–34	35–49	50–64	65+	All
*Annuitized wealth with heterogeneous returns & 150% real estate wealth (baseline)*					
% obs. with y $< 0$	9.8	9.5	6.2	1.4	6.8
% obs. with y $= 0$	15.7	9.2	8.3	6.4	10.7
Mean (in CHF)	1,848	10,866	23,013	55,379	20,786
Adj. Gini*	0.899	0.801	0.795	0.793	0.862
Top 1% share	0.433	0.343	0.337	0.347	0.412
*Annuitized wealth with constant returns & 150% real estate wealth*					
% obs. with y $< 0$	7.6	8.3	5.4	1.1	5.7
% obs. with y $= 0$	12.6	6.7	6.2	5.7	8.6
Mean (in CHF)	1,502	7,408	17,713	50,994	17,543
Adj. Gini*	0.808	0.775	0.772	0.781	0.855
Top 1% share	0.306	0.314	0.318	0.351	0.416
*Annuitized wealth with 100% real estate wealth & heterogeneous returns*					
% obs. with y $< 0$	11.3	16.8	12.0	2.1	10.7
% obs. with y $= 0$	15.7	9.2	8.3	6.4	10.7
Mean (in CHF)	1,358	7,287	16,654	45,650	16,078
Adj. Gini*	0.916	0.891	0.859	0.802	0.896
Top 1% share	0.503	0.456	0.405	0.364	0.460
*Labor income without self-empl. income*					
% obs. with y $< 0$	0.0	0.0	0.0	0.0	0.0
% obs. with y $= 0$	6.0	6.6	7.7	3.2	6.3
Mean (in CHF)	42,038	54,020	49,918	32,398	44,228
Adj. Gini*	0.334	0.339	0.384	0.293	0.375
Top 1% share	0.032	0.049	0.056	0.051	0.052

*Gini coefficient adjusted for negative values and normalized to $\left[ 0,1\right]$-range (see Chen et al. 1982)

**Table 8:** Alternative definitions of joint income-wealth by age group

	20–34	35–49	50–64	65+	All
*Baseline: Heterogeneous r., 150% real estate w., lab. inc. with 2/3 of self-empl. inc.*					
% obs. with y $< 0$	0.2	0.2	0.2	0.1	0.2
% obs. with y $= 0$	3.0	2.4	2.1	2.7	2.7
Mean (in CHF)	44,528	68,205	77,266	88,571	67,302
Adj. Gini*	0.335	0.343	0.422	0.562	0.448
Top 1% share	0.042	0.083	0.125	0.226	0.145
*Constant returns, 150% real estate wealth, lab. inc. with 2/3 of self-empl. inc.*					
% obs. with y $< 0$	0.1	0.2	0.2	0.1	0.1
% obs. with y $= 0$	2.8	2.0	1.7	2.4	2.4
Mean (in CHF)	44,182	64,747	71,966	84,185	64,060
Adj. Gini*	0.329	0.323	0.394	0.543	0.427
Top 1% share	0.037	0.068	0.106	0.224	0.132
*100% real estate wealth, constant returns, lab. inc. with 2/3 of self-empl. inc.*					
% obs. with y $< 0$	0.2	0.2	0.3	0.1	0.2
% obs. with y $= 0$	3.0	2.4	2.1	2.7	2.7
Mean (in CHF)	44,038	64,626	70,907	78,842	62,594
Adj. Gini*	0.333	0.339	0.414	0.542	0.434
Top 1% share	0.041	0.083	0.123	0.222	0.138
*Labor inc. w/o self-empl. inc., heterogeneous returns, 150% real estate wealth*					
% obs. with y $< 0$	0.3	0.6	0.6	0.1	0.4
% obs. with y $= 0$	3.0	2.5	2.2	2.7	2.8
Mean (in CHF)	43,886	64,886	72,932	87,777	65,014
Adj. Gini*	0.342	0.363	0.438	0.562	0.458
Top 1% share	0.042	0.084	0.128	0.227	0.147

*Gini coefficient adjusted for negative values and normalized to $\left[ 0,1\right]$-range (see Chen et al. 1982)

**Table 9:** Annuitized wealth and joint income-wealth by age group: Baseline imputation of age of married women assuming wives to be 2 years younger than husbands and alternative imputation assuming age-group varying age gaps*. All annuitized wealth is calculated with heterogeneous returns and adjusted real estate wealth

	20–34	35–49	50–64	65+	All
*Annuitized wealth with contant age gap of 2 years (baseline)*					
% obs. with y $< 0$	9.8	9.5	6.2	1.4	6.8
% obs. with y $= 0$	15.7	9.2	8.3	6.4	10.7
Mean (in CHF)	1,848	10,866	23,013	55,379	20,786
Adj. Gini*	0.899	0.801	0.795	0.793	0.862
Top 1% share	0.433	0.343	0.337	0.347	0.412
*Annuitized wealth with age-group varying age gaps**					
% obs. with y $< 0$	9.8	9.5	6.2	1.4	6.8
% obs. with y $= 0$	15.7	9.2	8.3	6.4	10.7
Mean (in CHF)	1,852	10,880	22,869	54,622	20,594
Adj. Gini*	0.899	0.801	0.795	0.794	0.861
Top 1% share	0.433	0.342	0.338	0.348	0.412
*Joint income-wealth with constant age gap of 2 years (baseline)*					
% obs. with y $< 0$	0.2	0.2	0.2	0.1	0.2
% obs. with y $= 0$	3.0	2.4	2.1	2.7	2.7
Mean (in CHF)	44,528	68,205	77,266	88,571	67,302
Adj. Gini*	0.335	0.343	0.422	0.562	0.448
Top 1% share	0.042	0.083	0.125	0.226	0.145
*Joint income-wealth with age-group varying age gaps**					
% obs. with y $< 0$	0.2	0.2	0.2	0.1	0.2
% obs. with y $= 0$	3.0	2.4	2.1	2.7	2.7
Mean (in CHF)	44,531	68,219	77,122	87,814	67,110
Adj. Gini*	0.335	0.343	0.422	0.561	0.447
Top 1% share	0.042	0.083	0.125	0.226	0.144

*20–34 years old: wives assumed to be 1 year younger, 35–49: 2 years younger, 50+: 3 years younger

## D Additional Quantile Plots

See Figs. 8, 9, 10 ,11 and 12

## E Additional Association Measures

In total, we show seven alternative association matrices in this Appendix for comparison to Fig. 3. Table 10 presents alternative associations measures.

- Figure 13 displays the relationship between current income and net wealth.
- Figures 14 and 15 display the relationship between labor income and capital income as defined by Atkinson and Lakner (2017), where income from self-employment is either split 2/3 to 1/3 between labor income and capital income or entirely included with capital income.
- Figure 16 differs from Figure 3 in that it uses a measure of annuitized wealth which does not inflate wealth by 150%.
- Figure 17 uses annuitized wealth with constant returns.
- Figure 18 uses the same variables as Figure 3 but only for one year.
- Figure 19 uses the same measure of annuitized wealth but a measure of labor income that excludes income from self-employment.

**Table 10:** Association between Labor Income & Annuitized Wealth by Variable Definition

	Baseline definition*	Annuitized wealth with constant returns	Annuitized wealth with 100% real real estate wealth	Labor inc. w/o self-empl. inc.
*Spearman’s Rank Correlation*				
All Observations	0.218	0.202	0.134	0.163
Ann. Wealth $>0$	0.155	0.128	0.118	0.092
Ann. Wealth $<0$	− 0.169	− 0.186	− 0.238	− 0.118
*Share of Jointly Affluent*				
% of Top Lab. Income 1% in Top Ann. Wealth 1%	12.5	11.7	13.1	10.5
% of Top Lab. Income 10% in Top Ann. Wealth 10%	19.9	17.9	19.0	17.1

* In the baseline definition, labor income includes 2/3 of self-empl. income and annuitized wealth is calculated with heterogeneous returns and with declared real estate wealth inflated by 150%. The first column in this table presents the association between these two variables. Remaining colums measure association with respect to alternative variable mentioned in title

## F Decomposition of Joint Income-Wealth

### Appendix F.A Copula Decomposition

To what extent is the distribution of joint income-wealth shaped by the two marginal distributions of labor income and annuitized wealth, respectively, and what is the role of the dependence among the two financial resources? To answer this question we borrow the idea of a decomposition proposed by Rothe (2015) who relates between-group differences in an outcome distribution to between-group differences in the marginal distributions of single covariates and to differences in the dependence among those covariates.

Consider the question more formally. Thanks to Sklar’s theorem we can express the distribution of joint income-wealth as

5 $$\begin{aligned} F_{Y_j}(y_j)&= \int \mathbbm {1}_{\{y_l + y_a \le y_j\}}dF_{Y_l,Y_a}(y_l,y_a) \nonumber \\&= \int \mathbbm {1}_{\{y_l + y_a \le y_j\}}dC_{Y_l,Y_a}(F_{Y_l}(y_l),F_{Y_a}(y_a)), \end{aligned}$$

with the distribution of labor income $F_{Y_l}(y_l)$, the distribution of annuitized wealth $F_{Y_a}(y_a)$, and the copula $C_{Y_l,Y_a}(\cdot ,\cdot )$ between the two marginals. $\mathbbm {1}_{\{\cdot \}}$ is an indicator function that equals to 1 if the statement is true and 0 otherwise. Following Rothe , this expression allows us to define counterfactual distributions that differ with respect to either single marginal distributions or the copula functions. With an appropriate counterfactual, we can capture the contribution of the single marginal distributions and their dependence structure to the overall level of joint income-wealth inequality.

Similar to scalar inequality measures, which define inequality in relation to perfect equality, we contrast the observed distribution to a hypothetical scenario under equality,

$$\begin{aligned} \Delta _{O}^{F(y)}=F_{Y_j}(y_j) - F^E_{Y_j}(y_j), \end{aligned}$$

where we would observe $F^E_{Y_j}(y_j) = \mathbbm {1}_{\{{\mathbb {E}}(Y_l)+{\mathbb {E}}(Y_a)\le y_l\}}$ if every individual received average labor income and average annuitized wealth. We first assess the role of the dependence structure. For this purpose, we introduce the counterfactual

$$\begin{aligned} F^{\text {ind}}_{Y_j}(y_j) = \int \mathbbm {1}_{\{y_l + y_a \le y_j\}}dC^{\text {ind}}_{Y_l,Y_a}(F_{Y_l}(y_l),F_{Y_a}(y_a)), \end{aligned}$$

where labor income and joint income-wealth are joined by the independence copula $C^{\text {ind}}(\cdot )$. The difference with respect to the observed distribution,

$$\begin{aligned} \Delta _{D}^{F(y)} = F_{Y_j}(y_j) - F^{\text {ind}}_{Y_j}(y_j), \end{aligned}$$

identifies the contribution of the dependence structure.

For the identification of the contribution of the single marginals, we define counterfactual distributions where there is either equality in labor income or equality in annuitized wealth. These counterfactuals correspond to the annuitized wealth distribution shifted by average labor income, $F_{Y_a}(y_j-{\mathbb {E}}(Y_l))$, and to the labor income distribution shifted by average annuitized wealth, $F_{Y_l}(y_j-{\mathbb {E}}(Y_a))$, respectively. By contrasting these counterfactuals to the distribution under independence,

$$\begin{aligned} \Delta _{M_l}^{F(y)}&= F^{\text {ind}}_{Y_j}(y_j) - F_{Y_a}(y_j-{\mathbb {E}}(y_l)), \\ \Delta _{M_a}^{F(y)}&= F^{\text {ind}}_{Y_j}(y_j) - F_{Y_l}(y_j-{\mathbb {E}}(y_a)), \end{aligned}$$

we distinguish the contribution of labor income and annuitized wealth, respectively. Finally, there is a potential interaction between the two marginals. We define this interaction effect as the remainder term

$$\begin{aligned} \Delta _{M_I}^{F(y)}&= F^{\text {ind}}_{Y_j}(y_j) - F^E_{Y_j}(y_j) - \Delta _{M_l}^{F(y)} - \Delta _{M_a}^{F(y)}, \end{aligned}$$

that is not explained by the ’direct’ contributions of labor income or annuitized wealth. By construction, the four effects add up to the aggregate difference between the observed distribution and the point mass distribution under equality, i.e.,

$$\begin{aligned} \Delta _{O}^{F(y)}&= \Delta _{D}^{F(y)} + \Delta _{M_l}^{F(y)} + \Delta _{M_a}^{F(y)} + \Delta _{M_I}^{F(y)}. \end{aligned}$$

The interaction effect is generally non-zero when distributional statistics are not a linear function of the underlying random variables. Assume for the sake of illustration that labor income $Y_l$ and annuitized wealth $Y_a$ follow a bivariate normal distribution with mean $\mu _l, \mu _a$, variance $\sigma ^2_l,\sigma ^2_a$, and correlation $\rho$, respectively. Suppose we are interested in decomposing quantile *p*. The quantile function of joint income-wealth would be $F_{Y_j}^{-1}(p)=(\mu _l + \mu _a) + \sqrt{\sigma ^2_l+\sigma ^2_a+2\rho \sigma _l\sigma _a}\Phi ^{-1}(p)$ with $\Phi ^{-1}(p)$, the quantile function of the standard normal distribution. When we set $\rho =0$, we get the counterfactual under independence, whereas quantiles under equality correspond to average joint income-wealth, $\left( F_{Y_j}^{E}\right) ^{-1}(p)=\mu _l+\mu _a$. Then, the decomposition terms are

$$\begin{aligned} \Delta _{D}^{F^{-1}(p)}&= \left( \sqrt{\sigma ^2_l+\sigma ^2_a+2\rho \sigma _l\sigma _a}-\sqrt{\sigma ^2_l+\sigma ^2_a}\right) \Phi ^{-1}(p) \\ \Delta _{M_l}^{F^{-1}(p)}&= \left( \sqrt{\sigma ^2_l+\sigma ^2_a} - \sigma _l\right) \Phi ^{-1}(p) \\ \Delta _{M_a}^{F^{-1}(p)}&= \left( \sqrt{\sigma ^2_l+\sigma ^2_a} - \sigma _a\right) \Phi ^{-1}(p) \\ \Delta _{M_I}^{F^{-1}(p)}&= \left( \sigma _a + \sigma _l - \sqrt{\sigma ^2_l+\sigma ^2_a}\right) \Phi ^{-1}(p), \end{aligned}$$

respectively. Since quantiles of the sum of two normally distributed variables are not a linear function of the distributional parameters, we get a non-zero interaction effect. However, when we decompose the variance we do not observe an interaction effect. In this case, the variance of “observed” joint income-wealth is $\sigma ^2_l + \sigma ^2_a + 2\rho \sigma _l\sigma _a$, while under perfect equality there is a variance of 0. The decomposition terms are

$$\begin{aligned} \Delta _{D}^{\text {Var}(y)}&= 2\rho \sigma _l\sigma _a \\ \Delta _{M_l}^{\text {Var}(y)}&= \sigma ^2_l \\ \Delta _{M_a}^{\text {Var}(y)}&= \sigma ^2_a \\ \Delta _{M_I}^{\text {Var}(y)}&= 0. \end{aligned}$$

As suggested by Rothe (2015, p. 329), we simulate the counterfactual distributions using the empirical marginal distribution functions. Concretely, we estimate the counterfactual distribution under independence by drawing a quasi-random sample of 100,000 independent quantile rank combinations and, then, computing the sum of the corresponding quantiles using the empirical quantile functions. Note that Rothe needs to model copulas since he is interested in decomposing *observed* between-group differences across distributions, e.g. wage change in the US between 1985 and 2005. In contrast, we do not need to estimate the copula of our observed distribution since we decompose a difference with respect to our constructed scenario under independence.

The remaining counterfactuals, where either labor income or annuitized wealth is equally distributed, are just the empirical distribution of the first income factor shifted by the sample mean of the other income factor. For the scenario under perfect equality, we just impute the sample mean. Distributional statistics and decomposition terms are estimated based on simulated data.

### Appendix F.B Gini Decomposition

We also perform a classical Gini decomposition by income source to complement the copula decomposition. This procedure identifies the contribution of each component to the Gini of joint income-wealth as the product of the component’s share in joint income-wealth, the component’s own Gini coefficient, and the component’s correlation with joint income-wealth ranks (see Lerman and Yitzhaki 1985). The third factor corresponds to the so-called “Gini correlation”, i.e., $R_l=\text {cov}(y_l,F(Y_j))/\text {cov}(y_l,F(Y_l))$ for labor income.

**Table 11:** Decomposition of Gini of Joint Income-Wealth by Source

	Labor Income	Annuitized Wealth*	Joint Income-Wealth
Income Share (*S*)	0.691	0.309	1.000
Gini of Source (*G*)	0.356	0.865	0.448
Correlation with Rank of JIW (*R*)	0.861	0.885	1.000
Share of Income Inequality ($I=S \cdot G \cdot R$)	0.211	0.236	0.448
Relative Income Inequality	0.472	0.528	1.000

*With heterogeneous returns

The results of this decomposition are displayed in Table 11. The decomposition confirms the main finding that annuitized wealth strongly contributes to inequality of joint income-wealth even though only a third of all joint income-wealth is annuitized wealth. It is the high inequality of annuitized wealth (Gini of 0.865) that leads to high levels of joint income-wealth inequality. Furthermore, the annuitized wealth ranks exhibt a strong correlation with joint income-wealth ranks (Gini correlation of 0.885). This correlation is also visible in Figs. 20 and 21 that show average labor income and annuitized wealth by joint income-wealth percentile for the entire population and the age groups, respectively. However, since the Gini correlation does not only measure the contribution of the copula between annuitized wealth and labor income but is also driven by the unequal distribution of annuitized wealth, we cannot conclude from the Gini decomposition that the dependence structure has high explanatory power for the aggregate inequality level of joint income-wealth.

## Footnotes

1 Statistics on the wealth and income distributions in Switzerland are available from the Swiss Federal Tax Administration at https://www.estv.admin.ch/estv/de/home/allgemein/steuerstatistiken/fachinformationen/steuerstatistiken.html. Across cantons, mean net wealth per household in 2015 ranges from 155,000 CHF in Jura to 1,139,000 CHF in Nidwalden and mean taxable income per household in 2015 ranges from 42,000 CHF in Valais to 95,000 CHF in Zug.

2 This is similar to the counting method by Alkire and Foster (2011a, 2011b), used in Peichl and Pestel (2013).

3 Note that capital gains are not directly observable in Swiss tax data because they are not taxed.

4 Between 2005 and 2015, same-sex couple could not formally marry in Switzerland but they could register which entails treatment equivalent to married couples in terms of taxation during the period under study. We only observe the gender of the “household head” and, therefore, treat all couples as one man and one woman.

5 Kopczuk (2015) provides an overview of other methods to measure wealth at the top of the distribution that are available in settings without a wealth tax. One is to use survey data such as the Survey of Consumer Finances (SCF) for the U.S. (e.g. Bricker et al. 2016; Rios-Rull and Kuhn 2016) or the SOEP for Germany (Frick et al. 2007). An alternative method, employed by Piketty and Zucman (2014) and Saez and Zucman (2016), is the capitalization method, which uses income tax data and imputes wealth from capital income flows. Notably, studies that rely upon the capitalization method tend to find greater wealth inequality than survey-based studies and conclude that inequality has been increasing more dramatically. Possible reasons for this discrepancy are the unclear distinction between labor and capital income and assumptions underlying the estimation of the capitalization factor, which links income flows to wealth stocks (Kopczuk 2015, 2016; Fagereng et al. 2020). A third method is to use estate tax multipliers (Kopczuk and Saez 2004) and a fourth is to use rich lists (Vermeulen 2018).

6 *Reinvermögen* is the only variable in our data that is truncated from below. The tax administrations of different Swiss cantons follow different procedures. The closely related paper by Krapf (2018), for example, uses a similar data set for the canton of Bern, in which raw net wealth is not truncated either from below or from above.

7 Note that real estate is the only item that is taxed based on location rather than based on residence. We, therefore, do not observe real estate owned by residents of the canton of Lucerne that is located outside the canton.

8 Pension savings can also be used before retirement to purchase owner-occupied housing or to start self-employment. Apart from these exceptions, pension plans are not directly marketable and cannot be used to finance current consumption. One can, therefore, argue even from a theoretical perspective that they are not wealth in a narrow sense. Saez and Zucman (2019), similarly, made the case for not including the present-value of future government transfers such as the public pension AHV.

9 Feld and Frey (2006) estimate income tax evasion to amount between 15 to 35 percent. We are not aware of any estimates of the extent of wealth tax evasion.

10 Lucerne’s tax law applies a 25% discount on the land register values, which already tend to be below market values, of owner-occupied housing. Moreover, agricultural land is assessed based on expected returns. However, our data do not allow us to distinguish between owner-occupied housing, agricultural land or other types of real estate assets.

11 Information on the age gap among couples in Switzerland is available from the Swiss Federal Statistical Office (FSO) at https://www.bfs.admin.ch/bfs/de/home/statistiken/bevoelkerung/familien/paare.html. At our request, the FSO compiles observed distributions of the gap for single cantons.

12 There are also 102,000 taxpayers in our data that are 19 years old or younger and 400 whose age is over 100. We keep those in our overall sample, but do not create separate age groups for them.

13 According to Bundesamt für Statistik (2021b), the average shares of the permanent population in Lucerne (Switzerland) over the period 2010 and 2015 were 21% (20%) for 20−34 years old individuals, 22% (23%) for 35–49 years old individuals, 19% (20%) for 50–49 years old individuals, and 16% (17%) for individuals aged 65 and older.

14 Unfortunately, we do not observe bequests. See Nekoei and Seim (forthcoming) for a recent study of how bequests affect wealth inequality in Swedish data.

15 See Martínez (2020) for a related comparison of the joint distribution of income and wealth across age groups.

16 In total, we show seven alternative association matrices in Appendix E for comparison to Figure 3. Figure 13 displays the relationship between current income and net wealth. Figures 14 and 15 display the relationship between labor income and capital income as defined by Atkinson and Lakner (2017), where income from self-employment is either split 2/3 to 1/3 between labor income and capital income or entirely included with capital income. Figure 16 differs from Fig. 3 in that it uses a measure of annuitized wealth which does not inflate wealth by 150% and Fig. 17 uses constant returns. Figure 18 uses the same variables as Fig. 3 but only for one year and Fig. 19 uses the same measure of annuitized wealth but a measure of labor income that excludes income from self-employment.

## References

[CR1] Aaberge R, Atkinson AB, Königs S (2018). From classes to copulas: Wages, capital, and top incomes. The Journal of Economic Inequality.

[CR2] Alkire S, Foster J (2011). Counting and multidimensional poverty measurement. Journal of Public Economics.

[CR3] Alkire S, Foster J (2011). Understandings and misunderstandings of multidimensional poverty measurement. The Journal of Economic Inequality.

[CR4] Alstadsæter, A., Martin, J., Wojciech, K., & Kjetil, T. (2016). “Accounting for Business Income in Measuring Top Income Shares: Integrated Accrual Approach Using Individual and Firm Data from Norway,” Discussion Paper 11671, C.E.P.R.

[CR5] Alstadsæter A, Johannesen N, Zucman G (2019). Tax Evasion and Inequality. American Economic Review.

[CR6] Atkinson, A. B. & Christoph, L. (2017). “Capital and labor : The factor income composition of top incomes in the United States, 1962–2006,“ Policy Research Working Paper 8268, The World Bank.

[CR7] Azpitarte F (2012). Measuring poverty using both income and wealth: A Cross-Country Comparison between the U.S. and Spain. Review of Income and Wealth.

[CR8] Bach L, Calvet LE, Sodini P (2020). Rich pickings? Risk, return, and skill in household wealth. American Economic Review.

[CR9] Bartels, C., & Katharina, J. (2015). “The Role of Capital Income for Top Incomes Shares in Germany,” Working Paper 2015/01, World Inequality Lab.

[CR10] Baselgia, E., & Martínez, I. Z. (2020). “A Safe Harbor: Wealth-Income Ratios in Switzerland over the 20th Century and the Role of Housing Prices,” Working Paper 2020/28, World Inequality Lab.

[CR11] Benhabib J, Bisin A (2018). Skewed wealth distributions: Theory and Empirics. Journal of Economic Literature.

[CR12] Berger D, Guerrieri V, Lorenzoni G, Vavra J (2018). House prices and consumer spending. Review of Economic Studies.

[CR13] Berman, Y., & Milanovic, B. (2020). “Homoploutia: Top Labor and Capital Incomes in the UnitedStates, 1950–2020,” Working Paper 2020/27, World Inequality Lab.

[CR14] Bourguignon F (2011). Non-anonymous growth incidence curves, income mobility and social welfare dominance. The Journal of Economic Inequality.

[CR15] Brandolini A, Magri S, Smeeding TM (2010). Asset-based measurement of poverty. Journal of Policy Analysis and Management.

[CR16] Bricker J, Henriques A, Krimmel J, Sabelhaus J (2016). Measuring income and wealth at the top using administrative and survey data. Brookings Papers on Economic Activity.

[CR17] Brown JR, Kapteyn A, Luttmer EFP, Mitchell OS, Samek A (2021). Behavioral impediments to valuing annuities: Evidence on the effects of complexity and choice bracketing. The Review of Economics and Statistics.

[CR18] Brülhart M, Dupertuis D, Moreau Elodie (2018). Inheritance flows in Switzerland, 1911–2011. Swiss Journal of Economics and Statistics.

[CR19] Brülhart, M., Gruber, J., Krapf, M., & Schmidheiny, K. (forthcoming).“Behavioral Responses to Wealth Taxes: Evidence from Switzerland,” *American Economic Journal: Economic Policy*.

[CR20] Brülhart, M., Krapf, M., Schmidheiny, K. (2021). “Die steigende Vermögenskonzentration in der Schweiz ist grösstenteils hausgemacht,” https://www.batz.ch/2021/09/die-steigende-vermoegenskonzentration-in-der-schweiz-ist-groesstenteils-hausgemacht/.

[CR21] Bundesamt für Statistik, (2021). “Neurentenstatistik 2019,” https://www.bfs.admin.ch/bfs/de/home/statistiken/soziale-sicherheit/berichterstattung-altersvorsorge/neurentenstatistik.html. [Online; accessed 11 November 2021].

[CR22] Bundesamt für Statistik. (2021). “Ständige und nichtständige Wohnbevölkerung nach institutionellen Gliederungen, Geschlecht, Zivilstand und Altersklasse,” https://www.bfs.admin.ch/bfs/de/home/statistiken/bevoelkerung/stand-entwicklung/alter-zivilstand-staatsangehoerigkeit.assetdetail.18404679.html. [Online; accessed 11 November 2021].

[CR23] Bütler, M., & Ramsden, A. (2017). “How taxes impact the choice between an annuity and the lump sum at retirement,” Economics Working Paper 1701, University of St. Gallen.

[CR24] Chen C-N, Tsaur T-W, Rhai Tong-Shieng (1982). The Gini Coefficient and Negative Income. Oxford Economic Papers.

[CR25] Chetty R, Grusky D, Hell M, Hendren N, Manduca R, Narang J (2017). The fading American dream: Trends in absolute income mobility since 1940. Science.

[CR26] Cowell FA, van Kerm P (2015). Wealth Inequality: A Survey. Journal of Economic Surveys.

[CR27] Durán-Cabré, J. M., Esteller-Moré, A., & Mas-Montserrat, M. (2019). “Behavioural responses to the (re) introduction of wealth taxes. Evidence from Spain,” Working Paper 2019/04, IEB.

[CR28] Fagereng A, Guiso L, Malacrino D, Pistaferri L (2020). Heterogeneity and persistence in returns to wealth. Econometrica.

[CR29] Feld, L. P., & Frey, B. S. (2006). “Tax evasion in Switzerland: The roles of deterrence and tax morale,” In: Nicolas Hayoz and Simon Hug, (eds.), *Tax Evasion, Trust and State Capacities*, Verlag Peter Lang, Bern, pp. 123–153.

[CR30] Fisher, J. D., Johnson, D. S., Smeeding, T. M., & Thompson, J. P. (2017). “Inequality in 3D: Income, consumption, and wealth,” Working paper series, Washington Center Equitable Growth for Equitable Growth.

[CR31] Foellmi, R., & Martínez, I. Z. (2017). “Die Verteilung von Einkommen und Vermögen in der Schweiz,” Public Paper 6, UBS International Center of Economics in Society.

[CR32] Frick, J. R., Grabka M., & Sierminska, E. M. (2007). “Representative Wealth Data for Germany from the German SOEP: The Impact of Methodological Decisions around Imputation and the Choice of the Aggregation Unit,” Discussion Paper 672, DIW Berlin.

[CR33] Halvorsen E, Thoresen TO (2021). Distributional effects of the wealth tax under a lifetime-dynastic income concept. Scandinavian Journal of Economics.

[CR34] Jann, B., & Fluder, R. (2015). “Erbschaften und Schenkungen im Kanton Bern, Steuerjahre 2002 bis 2012,” Working Paper 11, University of Bern Social Sciences.

[CR35] Jäntti, M., Sierminska, E. M., & Van Kerm, P. (2015). “Modeling the Joint Distribution of Income and Wealth,” In “Measurement of Poverty, Deprivation, and Economic Mobility,” Vol. 23 of *Research on Economic Inequality*, Emerald Publishing Ltd, pp. 301–327.

[CR36] Kennickell, A. B. (2009). “Ponds and Streams: Wealth and Income in the U.S., 1989 to 2007,” Finance and Economics Discussion Series 13, Federal Reserve Board.

[CR37] Kohli R (2017). Sterbetafeln für die Schweiz 2008/2013.

[CR38] Kopczuk W (2015). What Do We Know about the Evolution of Top Wealth Shares in the United States?. Journal of Economic Perspectives.

[CR39] Kopczuk W (2016). Comment on Bricker et al.: Measuring income and wealth at the top using administrative and survey data. Brookings Papers on Economic Activity.

[CR40] Kopczuk W, Saez E (2004). Top Wealth Shares in the United States, 1916–2000: Evidence From Estate Tax Returns. National Tax Journal.

[CR41] Krapf, M. (2018). “The Joint Distribution of Wealth and Income Risk: Evidence from Bern,” Working Paper 7130, CESifo.

[CR42] Kuypers S, Marx I (2018). Estimation of joint income-wealth poverty: A sensitivity analysis. Social Indicators Research.

[CR43] Lerman RI, Yitzhaki S (1985). Income Inequality Effects by Income Source: A New Approach and Applications to the United States. The Review of Economics and Statistics.

[CR44] Martínez, I. Z. (2020). Evidence from Unique Swiss Tax Data on the Composition and Joint Distribution of Income and Wealth. In Raj Chetty, John N. Friedman, Janet C. Gornick, Barry Johnson, & Arthur Kennickell (Eds.), *Measuring and Understanding the Distribution and Intra/Inter-Generational Mobility of Income and Wealth. *NBER Book Series Studies in Income and Wealth, Chicago: University of Chicago Press.

[CR45] McGrattan, E. R. (2015). “Taxing Wealth,” Economic Policy Paper 15-4, Federal Reserve Bank of Minneapolis.

[CR46] Moser, P. (2019). “Vermögensentwicklung und -mobilität: Eine Panelanalyse von Steuerdaten des Kantons Zürich 2006–2015,” statistik.info 02, Statistisches Amt Kanton Zürich.

[CR47] Nekoei, A., & Seim, D. (forthcoming). “How Do Inheritances Shape Wealth Inequality? Theory and Evidence from Sweden,” *Review of Economic Studies*.

[CR48] Pashchenko S (2013). Accounting for non-annuitization. Journal of Public Economics.

[CR49] Peichl A, Pestel N (2013). Multidimensional Well-Being at the Top: Evidence for Germany. Fiscal Studies.

[CR50] Piketty T, Zucman G (2014). Capital is Back: Wealth-Income Ratios in Rich Countries 1700–2010. The Quarterly Journal of Economics.

[CR51] Piketty T, Saez E, Zucman G (2018). Distributional national accounts: Methods and estimates for the United States. The Quarterly Journal of Economics.

[CR52] R Core Team. (2021). *R: A Language and Environment for Statistical Computing (Version 4.1.0)*, Vienna, Austria: R Foundation for Statistical Computing.

[CR53] Rendall MS, Speare A (1993). Comparing economic well-being among Elderly Americans. Review of Income and Wealth.

[CR54] Rios-Rull J-V, Kuhn M (2016). 2013 Update on the US earnings, income, and wealth distributional facts: A View from Macroeconomics. Quarterly Review, Federal Reserve Bank of Minneapolis.

[CR55] Roine J, Waldenström D (2008). The evolution of top incomes in an egalitarian society: Sweden, 1903–2004. Journal of Public Economics.

[CR56] Roller, M., & Schmidheiny, K. (2016). “Effective Tax Rates and Effective Progressivity in a Fiscally Decentralized Country,” Working Paper 5834, CESifo.

[CR57] Rothe C (2015). Decomposing the composition effect: The role of covariates in determining between-group differences in economic outcomes. Journal of Business and Economic Statistics.

[CR58] Saez E, Zucman G (2016). Wealth Inequality in the United States since 1913: Evidence from Capitalized Income Tax Data. The Quarterly Journal of Economics.

[CR59] Saez E, Zucman G (2019). Progressive Wealth Taxation. Brookings Papers on Economic Activity.

[CR60] Schweizerische Nationalbank. (2021). “Jährliche Bankenstatistik, Durchschnittliche Verzinsung ausgewählter Bilanzpositionen, Inland,” https://data.snb.ch/de/topics/ziredev#!/cube/ziverza. [Online; accessed 3 September 2021].

[CR61] Short, K. & Ruggles, P. (2005). “Experimental Measures of Poverty and Net Worth: 1996,” *Journal of Income Distribution*, *13* (3-4).

[CR62] StataCorp, Stata Statistical, and Software (2021). *Release 17, College Station*. TX: StataCorp LLC.

[CR63] Stutz, H., Bauer, T., & Schmugge, S. (2007). *Erben in der Schweiz—Eine Familiensache mit volkswirtschaftlichen Folgen.*, Zürich/Chur: Verlag Rüegger.

[CR64] Tang N, Mitchell OS, Mottola GR, Utkus SP (2010). The efficiency of sponsor and participant portfolio choices in 401(k) plans. Journal of Public Economics.

[CR65] van den Bosch,. (1998). Karel, “Poverty and Assets in Belgium,.” *Review of Income and Wealth**44*(2), 215–228

[CR66] Vermeulen P (2018). How fat is the top tail of the wealth distribution?. Review of Income and Wealth.

[CR67] Weisbrod BA, Hansen WL (1968). An income-net worth approach to measuring economic welfare. The American Economic Review.

[CR68] Wolff EN, Zacharias A (2009). Household wealth and the measurement of economic well-being in the United States. The Journal of Economic Inequality.

[CR69] Wüest, P. (2021). “Angebotspreisindex,” https://www.wuest.io/online_services_classic/angebotspreisindex/index.phtml. [Online; accessed 3 September 2021].

[CR70] Zagorsky, J. L. (2005). “Measuring Poverty Using Both Income and Wealth,” *Journal of Income Distribution*, *13*, (3–4).

